# In Situ Stress and Strain Detection in Lithium-ion Batteries: A Review of Optical, Acoustical, and Electrical Techniques

**DOI:** 10.1007/s40820-026-02275-9

**Published:** 2026-07-13

**Authors:** Xing’an Luo, Yueying Zhang, Yingjian Yu, Wenbo Liu

**Affiliations:** 1 College of Physics Science and Technology, Kunming University, Kunming, 650214 People’s Republic of China; 2 School of Mechanical Engineering, Sichuan University, Chengdu, 610065 People’s Republic of China

**Keywords:** Lithium-ion battery, In situ detection, Strain, Stress evolution, Volume expansion

## Abstract

Lithium-ion batteries (LIBs) have emerged as the key devices for energy storage, powering a global shift toward electrification in transportation and portable electronics. However, the relentless pursuit of higher energy densities for LIBs is fundamentally challenged by chemo-mechanical degradation, a process during which electrodes undergo volume changes during cycling, which in turn precipitates internal stress and limits battery life span. While the importance of these chemo-mechanical phenomena is widely recognized, a comprehensive review that systematically categorizes the diverse landscape of in situ detection techniques for stress and strain detection has been conspicuously absent. Herein, we address this gap by introducing a framework that organizes these techniques based on their physical modality: optical, acoustic, and electrical. Within this framework, we critically evaluate key methods, including optical laser beam position detection and multi-beam optical stress sensor, acoustic ultrasonic wave and acoustic emission, and electrical resistance strain gauge and in situ expansion meter. For each method, we detail its working principle, compare its advantages, and showcase key research applications that provide insight into improving battery performance. Finally, we outline the main challenges and future directions for in situ stress and strain detection in lithium-ion batteries.

## Highlights

- Establishing a three-dimensional classification and comparison framework based on physical modalities.
- In-depth analysis of signal interference and decoupling bottlenecks, unveiling commercialization engineering obstacles.
- Systematically outlining the specific technical path of AI empowering smart batteries.

## Graphical abstract

**Figure:**
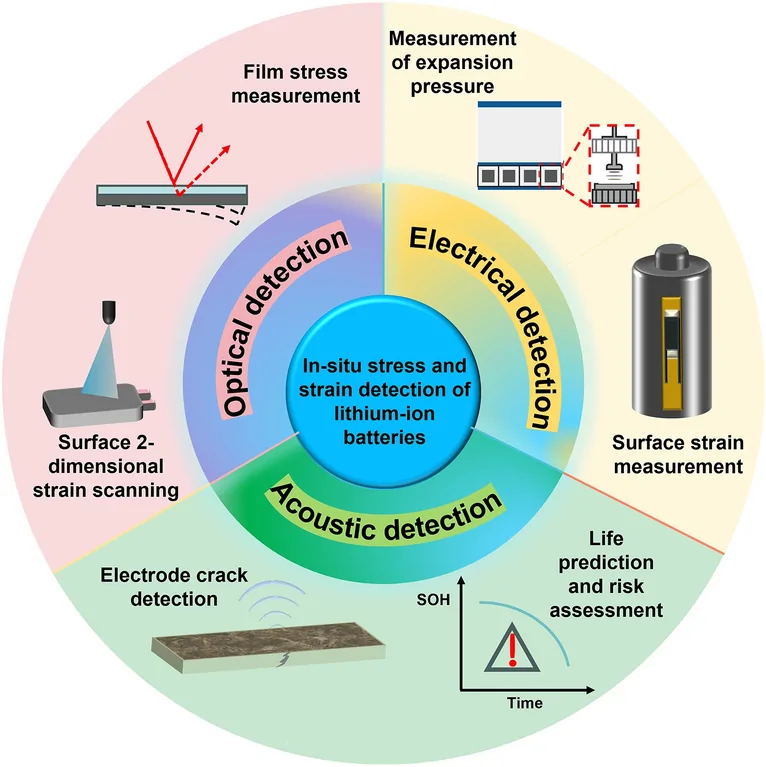

## Introduction

The global transition to sustainable energy is critically dependent on advanced battery technologies [1–3], among which lithium-ion batteries (LIBs) have emerged as the predominant energy storage solutions. Their ascendancy is driven by a combination of high energy density [2, 4, 5], long cycle life [6, 7], and favorable environmental compatibility [2, 4, 8–11], making them indispensable for applications from portable electronics to electric vehicles.

However, the capacity fading and safety risks associated with LIBs during prolonged cycling are closely linked to the deterioration of mechanical properties induced by internal stress. (When an external force acts on the electrode material, the internal particles will generate mutual interaction forces. The internal force per unit area borne by the electrode interior is expressed by stress.) This stress originates from the fundamental chemo-mechanical coupling in active materials, with lithium-ion intercalation and deintercalation upon cycling directly inducing significant lattice expansion and contraction. Such repetitive volume variations promote the progressive accumulation of mechanical stress, leading to structural degradation and dimensional instability within the electrode. This chemo-mechanical stress field is further complicated by interfacial dynamics at the SEI, internal pressurization from gas evolution, and abrupt phase transitions [12–16], and its detrimental effects span multiple scales: from atomic-level lattice distortions to mesoscopic nanoparticle fracture and macroscopic electrode swelling.

Computational simulations provide an indispensable theoretical framework for exploring the intrinsic connection between stress evolution and electrode microstructure. Diverse models, such as the phase field analysis model, have been developed to describe the coupling of lithium diffusion and stress, and to predict phenomena like crack propagation [17–19]. A notable example is the study by Hao et al., which used modeling to show that engineered surface tension in nanowire electrodes can generate a beneficial compressive stress field, thereby suppressing the internal tensile stress that drives fracture [20]. However, computational models are fundamentally constrained by the immense difficulty of integrating multi-scale physics. More crucially, their practical reliability demands validation against robust experimental data obtained under realistic operating conditions, highlighting a significant gap that current modeling efforts alone cannot bridge.

While conventional experimental in situ characterization techniques like scanning electron microscopy (SEM) [21, 22], transmission electron microscopy (TEM) [23, 24], and atomic force microscopy (AFM) [25–29] provide vital postmortem evidence of mechanical failure (e.g., particle fragmentation and structural collapse), they are fundamentally incapable of capturing the dynamic processes within a cell. Their reliance on battery disassembly confines them to static analysis and risks introducing preparation-induced artifacts. Therefore, to overcome the fundamental limitations of both theoretical simulations and post-event analysis, it is imperative to employ advanced, in situ, and nondestructive techniques that can probe chemo-mechanical phenomena directly within operating LIBs.

**Fig. 1:**
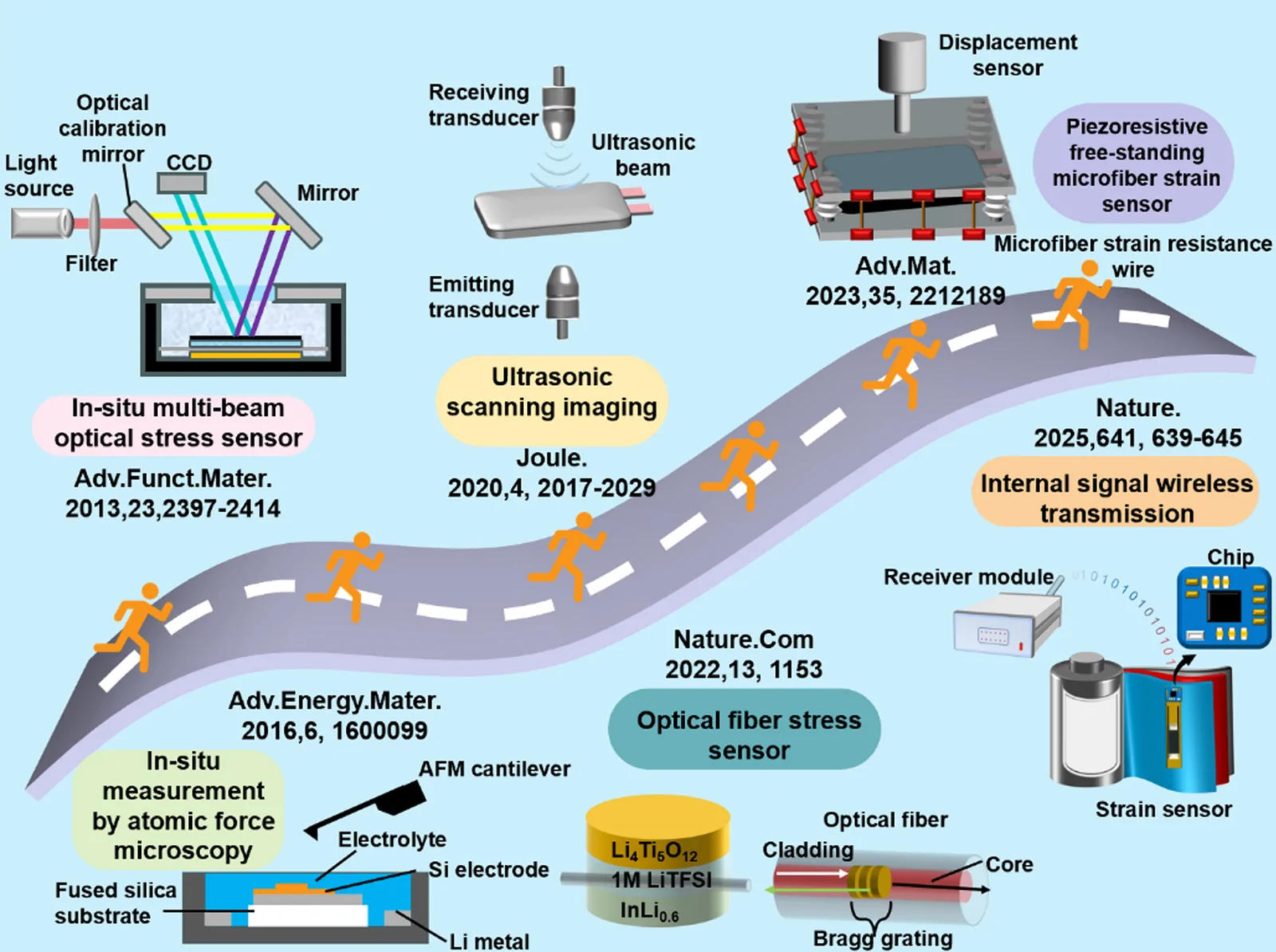
Development history of in situ stress and strain detection methods for lithium-ion batteries

The field of in situ chemo-mechanical detection research for LIBs has recently undergone rapid evolution, marked by the development of novel techniques that enable unprecedented insights into battery. Figure 1 provides a comprehensive roadmap of this progress, charting the key technological milestones that are driving the field toward greater practical implementation [15, 30–34]. This progress is characterized by several key trends, including internalization, multi-dimensionality, high precision, and intelligent integration. However, the field currently lacks a systematic synthesis that classifies and critically evaluates the burgeoning array of in situ techniques for stress and strain (strain represents the relative deformation amount of the electrode, which is the rate of change in the length of the original electrode. It emphasizes the degree of deformation rather than the magnitude of the deformation) analysis, hindering a clear understanding of their relative strengths and weaknesses.

**Fig. 2:**
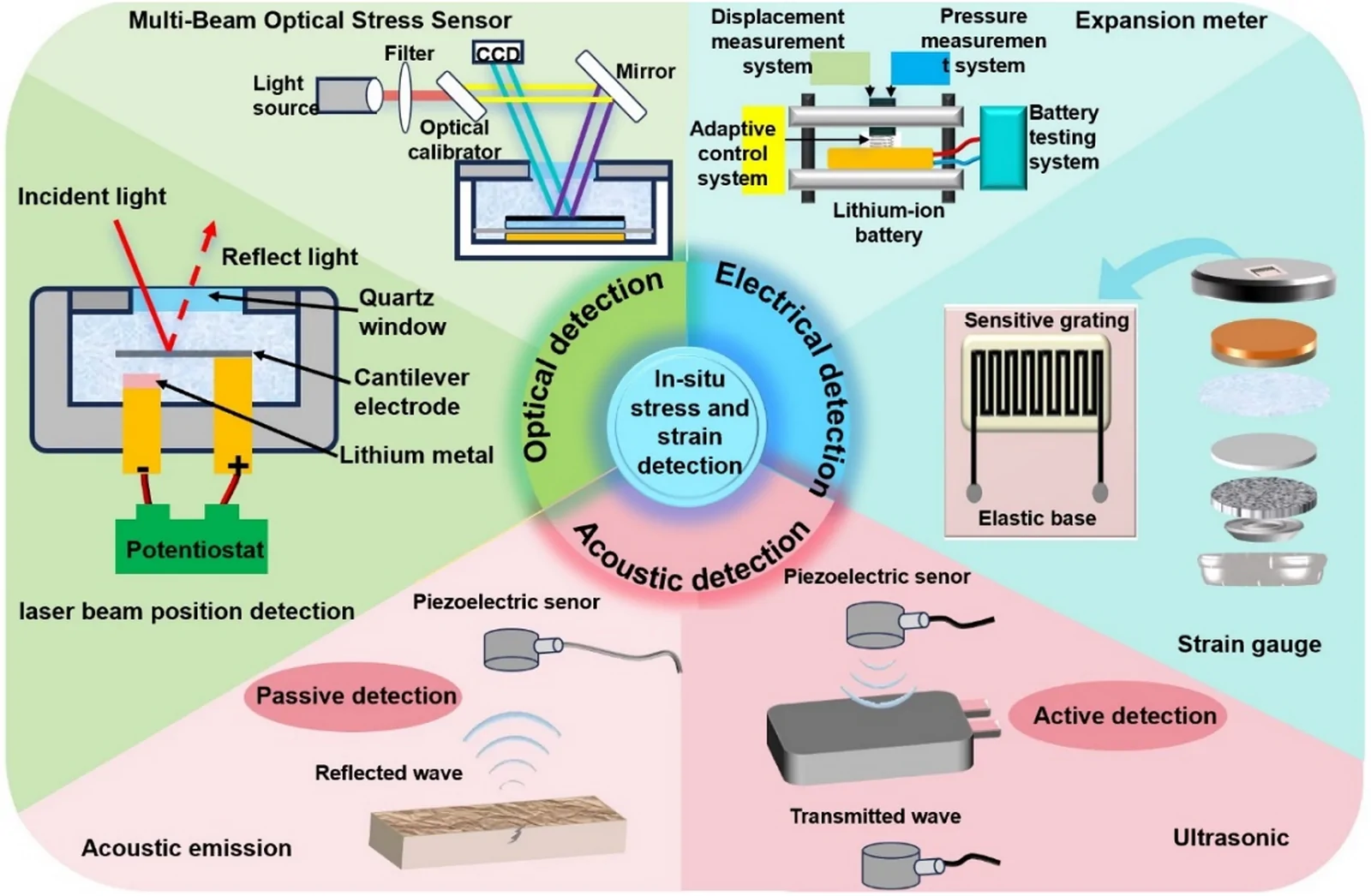
Schematic diagram of in situ stress and strain measurement of lithium-ion batteries

This review first systematically classifies and discusses the sources of stress in LIB electrode materials. At the same time, based on the fundamental physical conversion principles of in situ detection—optics, acoustics, and electricity (Fig. 2)—the most advanced in situ stress and strain detection methods are classified, thereby directly bridging a critical gap in the literature: the lack of a unified, principle-based framework for evaluating in situ monitoring techniques. For each modality, we present a rigorous, cross-cutting analysis spanning fundamental operating mechanisms, representative implementations, performance trade-offs, and persistent challenges in real-world deployment.

## Evolution of Stress in Electrode Materials

### Origins of Stress in Electrode Materials

**Fig. 3:**
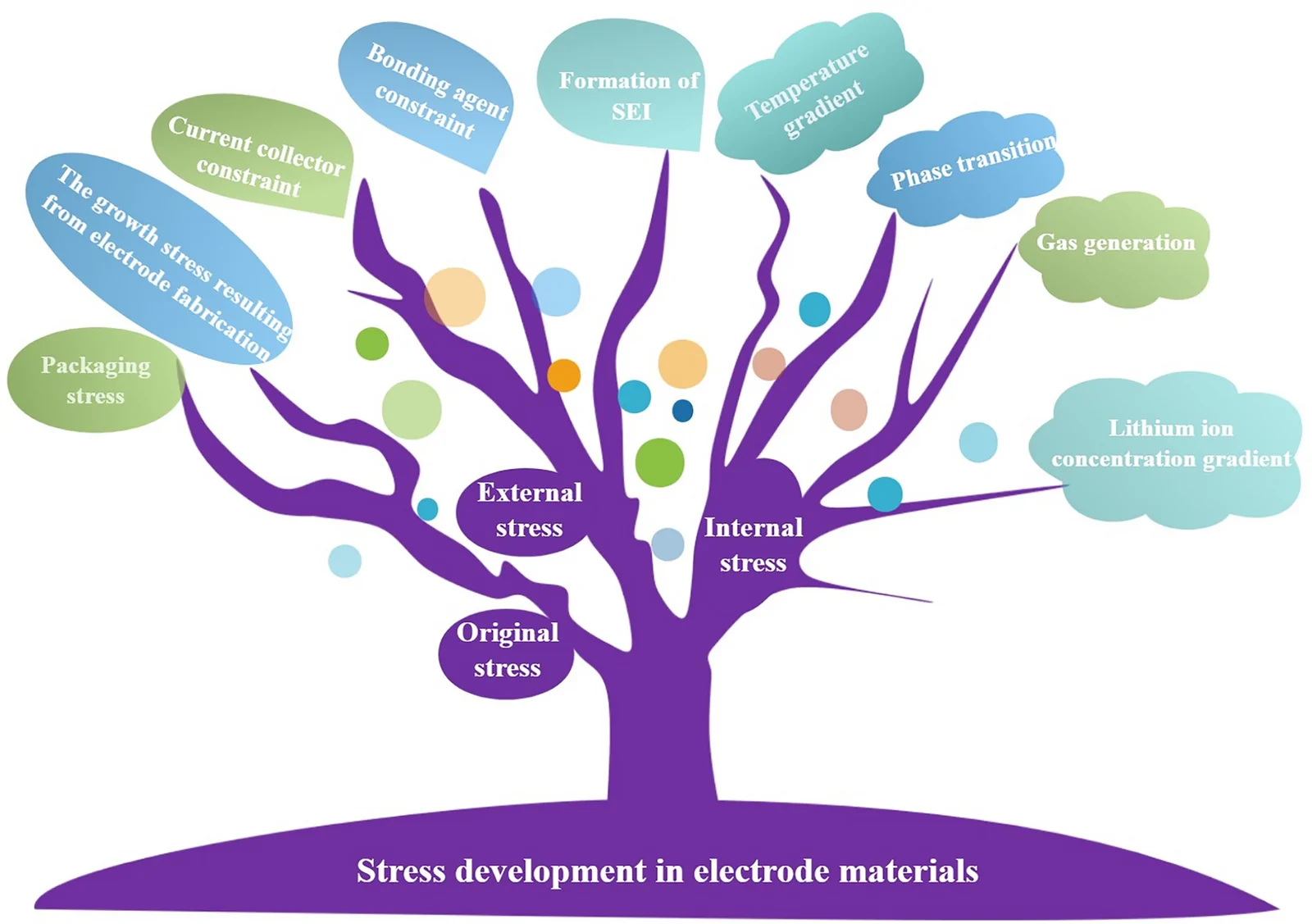
Origins of stress in electrode materials

The mechanical stress within electrode materials is a complex phenomenon arising from a confluence of factors. To systematically dissect this issue, we can classify these stress into three principal categories based on source of stress: internal stress, external stress, and “original stress” (Fig. 3). Internal stress is intrinsically generated by the electrochemical process within the active materials themselves. External stress arises from the mechanical constraints imposed by surrounding components in the battery configuration. Finally, “original stress,” more formally termed residual and assembly stress, is the stress state established during electrode preparation and cell assembly, existing even before the first cycle [35].

### Internal Stress

Internal stress originates from the physical and chemical changes within electrode materials during electrochemical cycling [36]. Its primary sources include: (1) phase transformation and diffusion. The most significant source is the volume changes associated with lithium insertion and extraction. First-order phase transitions induce large, localized stress at the phase boundary due to the molar volume mismatch between the coexisting phases [37]. For instance, Cheng et al. designed a three-dimensional porous silicon/carbon composite material (GaIn-Si@PCC) to alleviate the stress caused by lithiation and achieve crack self-healing during delithiation [14]. In addition, the H1 → HII phase transition in the LiCoO_2_ electrode is manifested as a stress plateau, suggesting an internal stress regulation mechanism in the electrode [38]. Consequently, diffusion-induced stress arises from lithium-ion concentration gradient, particularly during high-rate operation. This typically results in compressive stress on the surface and tensile stress in the interior. In silicon anodes, the stress can reach 1.2–2.5 GPa, leading the material beyond its elastic limit into viscous flow [13, 39–41]. (2) Gas generation and thermal effects. Parasitic reactions, such as electrolyte decomposition, can produce gas (e.g., H_2_, CO_2_, C_2_H_4_) within the sealed cell [3, 42]. The accumulation of this gas increases internal pressure, exerting a global compressive stress on the electrode stack [43]. Especially in the later stage of battery aging (SOH about 80%), it can lead to a thickness increase of 20–50% [44]. Additionally, Joule heating during cycling creates temperature gradients, and the thermal expansion difference induces thermal stress. Although it is relatively small under normal cycling conditions, it cannot be ignored under high-rate or thermal runaway conditions. Advanced optical fiber temperature sensors—such as ratiometric fluorescence fibers with sub-0.2 °C accuracy [45, 46]—have been developed to monitor internal temperature during cycling, and have revealed that single-crystalline Ni-rich cathodes generate substantially less polarization heat than polycrystalline counterparts [47, 48]. Not only that, the detection and analysis of gases inside batteries are also a special challenge faced by the future development of smart batteries. Tang et al. systematically summarized the latest progress and core applications of in situ differential electrochemical mass spectrometry technology in the study of gas generation in rechargeable batteries. For the first time, a complete knowledge chain from “gas detection–mechanism revelation–performance degradation–suppression strategy” was constructed, and multi-level gas suppression schemes based on electrolyte engineering, electrode interface regulation, and system compatibility optimization were proposed [49]. Recent operando studies employing embedded fiber bragg grating (FBG) sensors have provided deeper insights into the multi-component nature of stress generation. Zhang et al. distinguished two fundamentally different stress components in NCM811 cathodes: chemical stress, arising from reversible unit-cell volume changes during (de)lithiation, and structural stress, originating from the anisotropic lattice strain of randomly oriented primary particles within polycrystalline secondary particles [50]. Structural stress manifests as an anomalous stress increase during delithiation that is absent in single-crystalline NCM and ultimately drives micro-crack formation [51].

The consequences of these internal stresses are severe. It is a primary driver of mechanical degradation, including particle cracking, pulverization, and delamination from the conductive network, leading to capacity fade and impedance rise. Moreover, stress is fundamentally coupled with the electrochemical properties. Compressive stress generally reduces the driving force for lithiation, thereby raising the open-circuit potential and inhibiting the lithium-ion diffusion. Tensile stress has the opposite effect. This “stress–potential” and “stress–diffusion” coupling creates a dynamic feedback loop that directly influences the capacity, Coulombic efficiency, and voltage plateau.

### Electrode Material External Stress

**Fig. 4:**
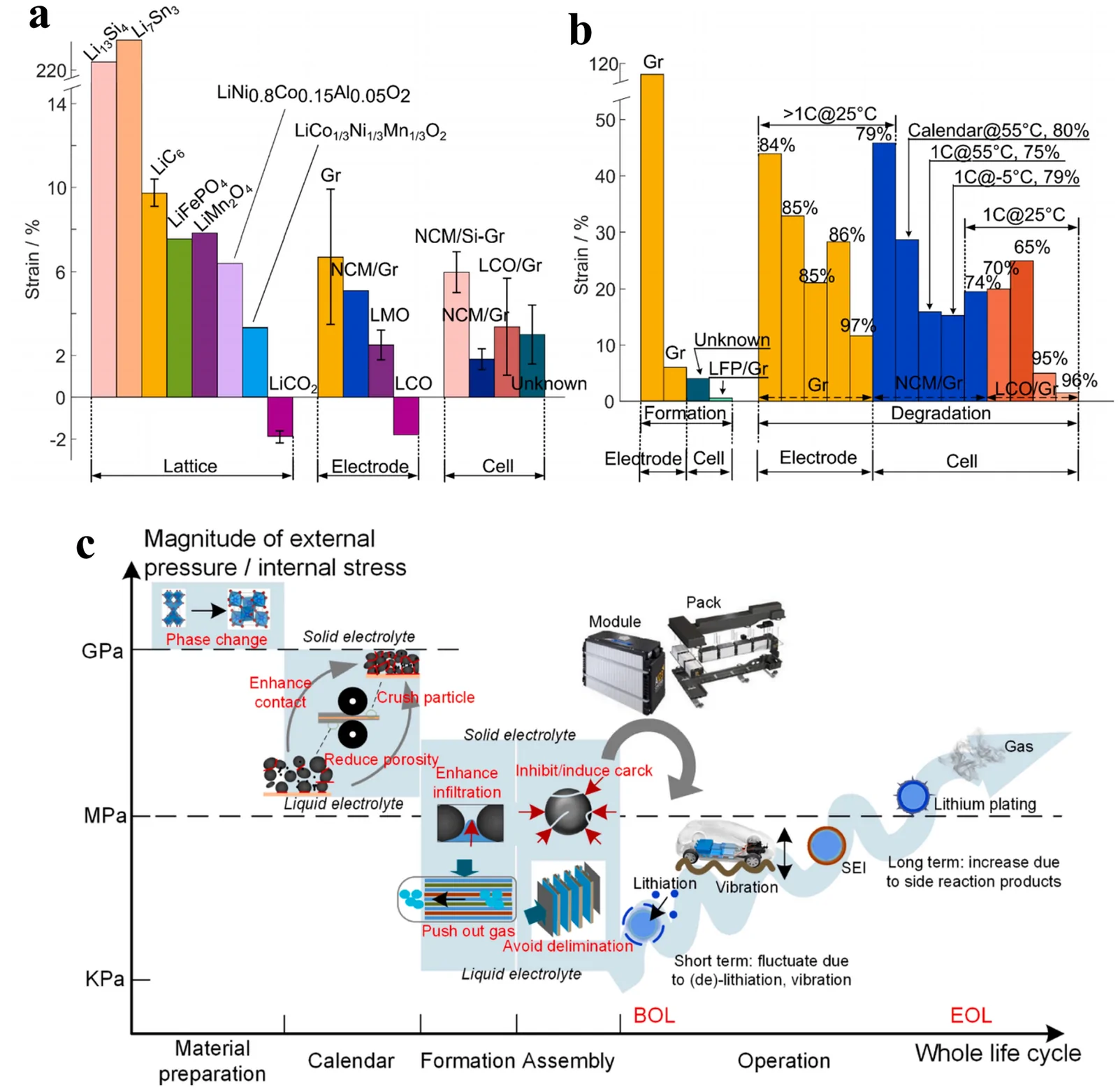
**a** Lithiumation-induced deformation and **b** side reaction-induced deformation of different electrode materials in lithium-ion batteries. Positive values indicate expansion, and negative values indicate contraction. The deformation of particles and electrodes only includes the deformation caused by lithiumation, while the deformation of the battery is the result of the combined effect of lithiumation-induced deformation and thermal-induced deformation. **c** Range of mechanical load intensities that batteries and their components undergo during the manufacturing process and operation. Here, BOL refers to the starting point of battery life span, and EOL refers to the end point of life span [55]. ©2022 Elsevier

External stress originates from the mechanical constraints imposed by other components in the electrode structure. The current collectors (such as Cu and Al foils) with high stiffness and low expansion rate strongly restrict the in-plane deformation of electrode materials, thereby inducing in-plane biaxial stress. For instance, the elastic modulus and distribution pattern of the binder directly affect the constraint strength on active particles. A binder with high stiffness, such as sodium alginate, induces approximately 150 MPa of compressive stress in a composite Si electrode [52]. In addition, the binder in the electrolyte will undergo swelling behavior. Sethuraman et al. measured through experimental tests long ago that this swelling behavior would add an extra compressive stress of approximately 2 MPa [53]. For instance, Nadimpalli et al. investigated the influence of the SEI layer on the overall compressive stress development during the lithiation process of thin-film silicon electrodes by monitoring the stress changes in the copper current collector caused by the formation or growth of the SEI layer. They found that a 100-nm SEI layer on the thin-film silicon electrode would generate a maximum stress of up to 240 MPa [54]. Li et al. summarized the deformation caused by lithiation and that caused by side reactions reported in previous research literature in Fig. 4a, b. They found that when the battery degraded to approximately 80% of its initial state, the deformation caused by side reactions could reach 20–50% [49].

Moderate external pressure can improve interlayer contact, suppress lithium dendrite growth, reduce interface impedance, and enhance cycling stability; however, excessive external pressure can lead to pore closure, a sharp increase in the ionic impedance of the separator, uneven local current density, and accelerated lithium deposition, thereby inducing internal short circuits and thermal runaway, and significantly shortening the battery’s life span. In terms of the coupling between external stress and electrochemical properties, external pressure directly affects the ionic and electronic transport impedance by altering the porosity, tortuosity, and contact state of the electrodes: Compression in the initial stage can reduce ohmic impedance, but excessive compression will cause the ionic impedance to increase exponentially. Additionally, the superposition of external pressure and internal stress will affect the growth stress and stability of the SEI layer: The irreversible compressive stress generated during SEI formation can change the local potential distribution on the electrode surface, thereby regulating the composition and thickness of the SEI, forming “stress–interfacial reaction” positive feedback. Therefore, the optimization of external stress is not only a mechanical design issue, but also a key means to extend battery life by regulating electrochemical kinetics.

### “Original Stress” of Electrode Material

During the battery packaging and initial activation stages, the initial stress mainly originates from the mechanical constraints of module assembly. When the electrode material are inserted into a hard shell or pouch and pre-tightening force is applied through bolts, end plates, etc., the cells undergo axial or radial compression, resulting in initial compressive stress within the electrode materials; the magnitude of this stress is closely related to the stiffness of the shell, the size of the pre-tightening force, and the buffer material [53, 56, 57].

During the electrode preparation stage, the initial stress mainly comes from thin-film deposition, rolling treatment, and binder swelling. For thin-film electrodes, the active materials are typically deposited on the current collector at temperatures above room temperature using gas-phase or electrochemical methods. During the cooling process, due to the mismatch in thermal expansion coefficients and lattice parameters between the material and the substrate, residual tensile or compressive stress is generated (which can be either tensile or compressive). For porous composite electrodes, after coating and drying, the electrode undergoes pressing treatment under tens of to hundreds of megapascals of linear pressure, resulting in particle rearrangement, fragmentation, and pore closure. After unloading, some of the compressive deformation is locked in, forming residual compressive stress **(**Fig. 4c**)**. Additionally, when the electrode contacts the electrolyte (such as during the filling stage), the polymer binder absorbs the solvent and undergoes volume expansion (the change in the occupation of the overall space). However, due to the mechanical constraints of the active particles and the current collector, it cannot expand freely, thereby inducing compressive stress within the electrode [58, 59]. These initial stresses already exist before the electrochemical cycling begins and directly affect the stress evolution and structural stability during the subsequent cycling.

## In Situ Optical Detection

Numerous in situ detection methods face limitations in real-time monitoring of electrode materials, and certain techniques exhibit inherent time delays. Owing to the propagation characteristics of light, optical detection methods enable rapid and efficient acquisition of dynamic in situ information from electrode materials. These techniques utilize signals generated through the interaction of photons with matter—such as scattering, diffraction, and interference—constituting a nondestructive, high-precision detection approach with strong resistance to magnetic interference. These distinctive advantages render optical methods particularly effective for investigating the structural evolution of electrode materials and the underlying microscopic reaction mechanisms, such as phase transformation-induced stress. With continuous advancements in detection methodologies, several optical detection techniques, such as the multi-beam optical stress sensor (MOSS) [15, 24, 54, 56, 60–74] and laser beam position detection (LBPD) methods, have been progressively adapted for in situ stress–strain analysis of LIBs electrode materials during cycling, which enable real-time monitoring of structural evolution and mechanical behavior (i.e., stress/strain) of internal electrode materials under operational conditions.

### Laser Beam Position Detection

**Fig. 5:**
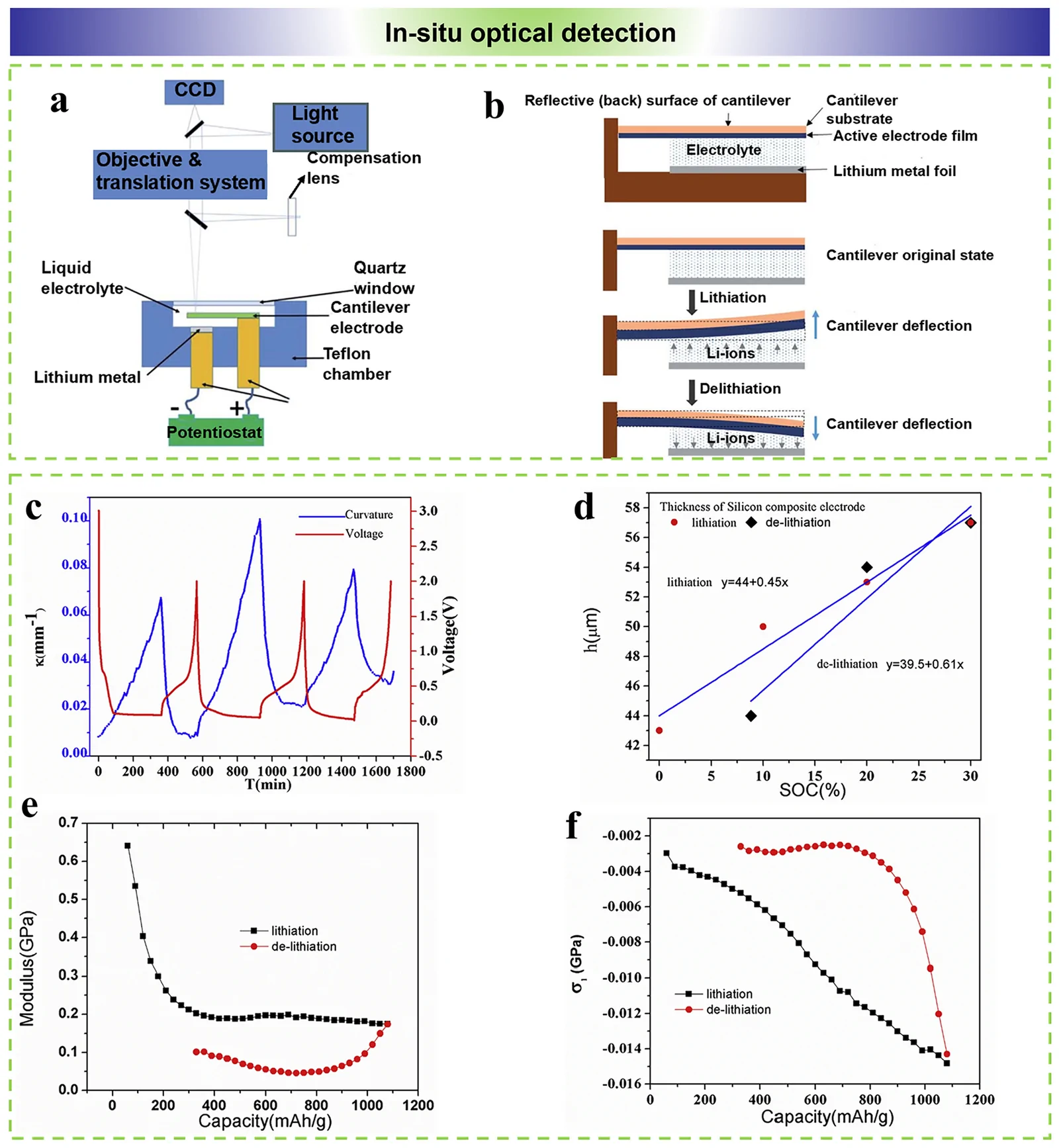
Schematic diagram of **a** the LBPD experimental setup and **b** the bending of the cantilever during the lithiation/delithiation process in LBPD [76]. ©2018 Pergamon-Elsevier Science Ltd. All rights are reserved. **c** Variation curves of voltage and curvature of Si/PVDF composite electrodes during the first three cycles of constant current (C/20) charge–discharge cycles. During the discharge process, lithium ions react with silicon and undergo deformation. As the deformation of the active layer increases, the electrode bends toward the current collector side, and the curvature gradually increases, the charging process is just the opposite. **d** Thickness variation of the composite electrode during the second cycle. **e** Elastic modulus of the composite electrode varies with the electrode capacity in the second cycle. **f** Stress evolution in the second cycle period [68]. ©2017 Elsevier

The LBPD method is a widely employed technique for real-time monitoring of stress evolution in thin-film electrodes. This approach enables stress measurement by detecting curvature changes in the electrode film adhered to a rigid substrate—the so-called film–substrate system—induced by mechanical stress. A typical experimental configuration employs a cantilever beam-structured electrode (Fig. 5a). The setup incorporates a custom-designed electrochemical cell containing a cantilever beam working electrode and a counter electrode, with an optical window integrated into the top to facilitate laser transmission while maintaining system hermeticity. To ensure measurement accuracy and prevent interference, the entire experiment is conducted within an inert atmosphere. As the active material film undergoes volume changes during cycling, the substrate constrains this strain into a measurable deflection of the cantilever. Specifically, volumetric expansion induces upward deflection, whereas contraction leads to downward bending (Fig. 5b). The curvature changes of the cantilever, induced by internal stress variations in the thin-film electrode, can be monitored in real time using laser reflection technology. This curvature change is determined by measuring the deflection (or positional shift) of a focused laser beam reflected from the back surface of the substrate, enabling the calculation of the stress evolution in the electrode material. The stress σ in the thin film is calculated from the measured substrate curvature, Stoney’s equation [75] (Eq. (1)).

1 $$\sigma =\frac{E{{h}_{s}}^{2}}{6(1-\nu ){h}_{f}R}$$

where *E* and ν are the Young’s modulus and Poisson’s ratio, respectively; ${h}_{s}$ and ${h}_{f}$ are the thickness of the substrate and active material thin film; and *R* represents the experimentally measured radius of curvature. It should be emphasized that the aforementioned equation is valid only under the condition that the thickness (${h}_{f}$) of the active material thin film is less than the substrate thickness (${h}_{s}$), and that both dimensions are significantly smaller than the in-plane characteristic length. Consequently, the applicability of this model is restricted to simplified electrode systems composed of non-porous and isotropic materials. For instance, Li et al. reported a method for measuring the curvature (Fig. 5c), elastic modulus (Fig. 5e), thickness (Fig. 5d), and stress evolution of planar electrodes by designing a cantilevered planar silicon electrode without holes and a quartz test cell. It was found that the modulus of the silicon electrode would decrease significantly during the lithiation process (approximately from 0.63 to 0.18 GPa), and a rapid decrease in compressive stress caused by crack formation was observed during the initial lithiation process (Fig. 5e, f) [68]. However, in most cases, the working electrode contains not only active materials but also conductive agents, binders, and the structure of the material itself, all of which can affect the curvature change of the electrode during lithiation and delithiation. Therefore, in order to better adapt to the measurement of the curvature of such composite electrodes, the original Stoney’s equation needs to be modified to calculate the stress evolution based on the curvature change [69, 70]. The modified form is

2 $$\sigma { = }\frac{{{\mathrm{M}}_{{\mathrm{s}}} {\mathrm{h}}_{{\mathrm{s}}} }}{{{\mathrm{6h}}_{{\mathrm{f}}} {\mathrm{Rf}}({\mathrm{h}}_{{\mathrm{i}}} ,{\mathrm{M}}_{{\mathrm{i}}} )}}$$

3 $$f(h_{i} ,M_{i} ) = 1 + \frac{{h_{f} }}{{h_{s} }}[1 + 4\frac{{h_{f} }}{{h_{s} }}\frac{{M_{f} }}{{M_{s} }} + 6\frac{{h_{f}^{2} }}{{h_{s}^{2} }}\frac{{M_{f} }}{{M_{s} }} + 4\frac{{h_{f}^{3} }}{{h_{s}^{3} }}\frac{{M_{f} }}{{M_{s} }} + \frac{{h_{f}^{4} }}{{h_{s}^{4} }}\frac{{M_{f}^{2} }}{{M_{s}^{2} }}]^{ - 1}$$

4 $$M_{f} = \frac{{E_{f} }}{{1 - \nu _{f} }} = \frac{{\phi _{{{\mathrm{electrode}}}} E_{{{\mathrm{electrode}}}} + \phi _{b} E_{b} + \phi _{c} E_{c} }}{{1 - (\phi _{{{\mathrm{electrode}}}} \nu _{{{\mathrm{electrode}}}} + \phi _{b} \nu _{b} + \phi _{c} \nu _{c} )}}$$

where *M*, *E*, ν, Φ, and D denote the biaxial modulus, Young’s modulus, Poisson’s ratio, and volume fraction, respectively, while the subscripts *s*, *f*, *b*, and *c* correspond to the substrate, active material film, binder, and conductive additive. The modified formulation provides a more accurate description of stress evolution in composite electrodes under the combined influence of multiple factors during electrochemical cycling. For example, Sethuraman et al. employed the wafer curvature method to bond graphite particles onto a silicon wafer using a PVDF (8%) binder and epoxy resin, which enabled the first real-time measurement of stress evolution in graphite anodes during electrolyte wetting and electrochemical cycling. This study not only revealed the characteristic stress evolution behavior of graphite electrodes but also demonstrated that the composite electrode develops approximately 1–2 MPa of compressive stress upon electrolyte wetting due to binder swelling [71].

However, the stress distribution is uneven during the volume shrinkage/expansion of the electrode material. The single-point measurement method used in the cantilever method is only suitable for measuring the average stress generated by the interaction between the substrate and the film, and cannot accurately reflect the overall internal stress state of the electrode material [72]. Furthermore, the method’s sensitivity to environmental disturbances presents a practical challenge. Environmental vibrations, for example, can cause the loss of measurement reference points or increase experimental errors. To mitigate these effects and ensure data validity, a stable environment, free from mechanical perturbations, is a critical prerequisite.

### Multi-beam Optical Stress Sensor

**Fig. 6:**
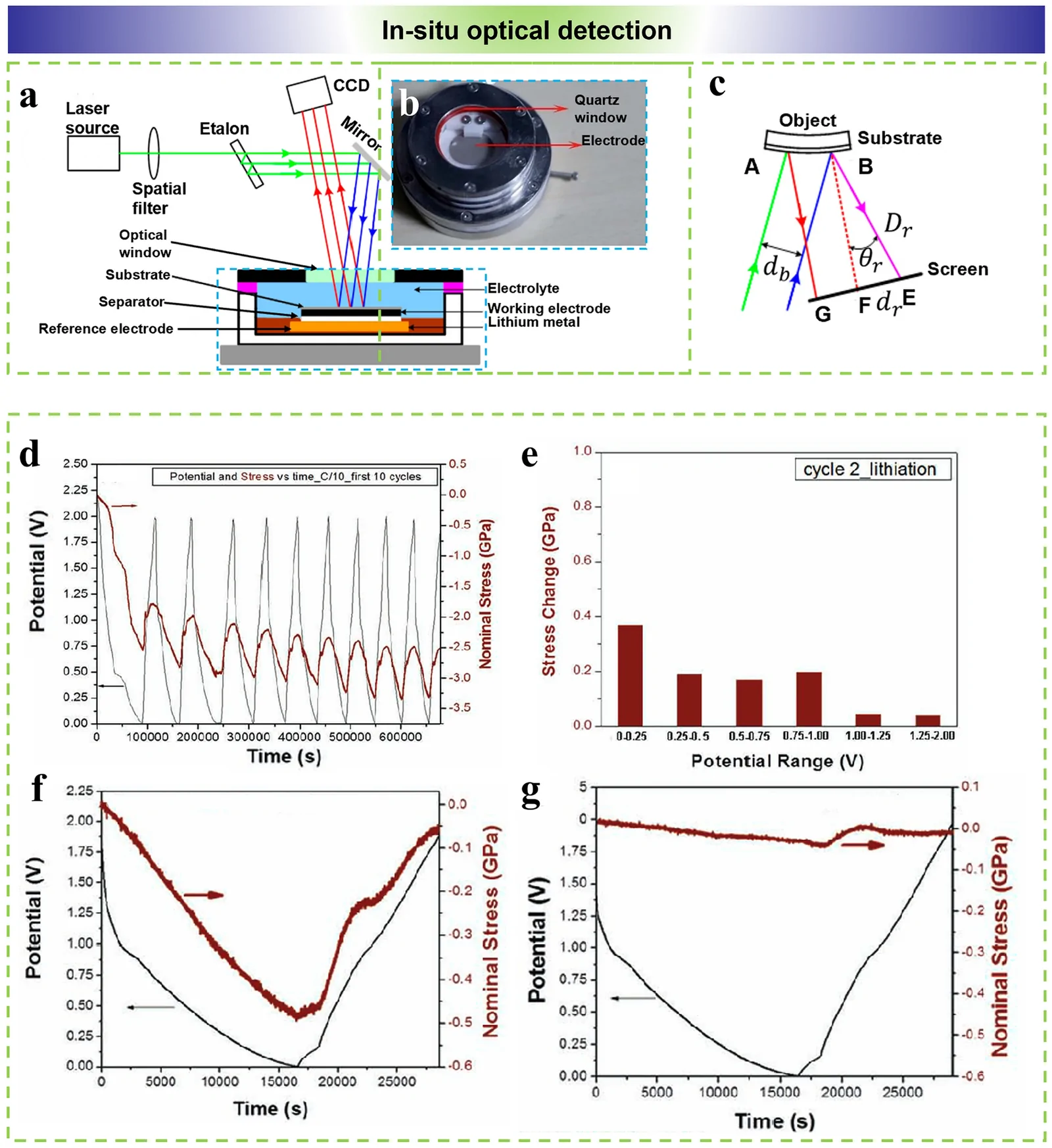
**a** Schematic diagram of MOSS experimental setup All rights are reserved. **b** Digital photo of the custom electrochemical cell [78]. ©2019 Royal Soc Chemistry. Schematic diagram of **c** the laser irradiation and substrate reflection principle of MOSS measurement technology [62, 74]. ©2010 Elsevier. ©2010 Elsevier Science Inc. **d** Variation of voltage and stress of graphene film electrode over time in the first 10 cycles at a constant rate of C/10. **e** Development of stress within different potential ranges in the second lithiation half-cycle under the same current conditions, starting from the second lithiation half-cycle, the stress inversion of the graphene film electrode is higher within the potential range of 0.25–0.5 V, and these differences are related to the crystal orientation of the graphene film. At a constant current rate of C/5, when the MOSS laser array is **f** perpendicular to the transverse direction of the graphene plane **g** parallel to the transverse direction of the graphene plane (reproduced with permission [15]. ©2013 Wiley-V C H Verlag Gmbh

The MOSS method is a high-precision optical technique used for in situ monitoring of internal stress in thin-film electrodes. Based on the real-time measurement of the curvature change of the electrode in the thin film–substrate structure, the internal stress variation of the thin film material is indirectly deduced. As shown in Fig. 6a, two parallel laser beams are projected onto the substrate surface of the working electrode, and the substrate curvature is measured in real time by the change in the distance between the reflected beams (Fig. 6b, c). The curvature radius of the electrode substrate changes due to internal stress (Fig. 6c):

5 $$R = \frac{{2D_{r} d_{b} }}{{d_{r} \cos \theta_{r} }}$$

Among them, ${D}_{\mathrm{r}}$ is the distance between the beam detector and the incident beam point on the substrate, ${d}_{\mathrm{b}}$ is the distance between the two incident beams, ${d}_{\mathrm{r}}$ is the distance between the two reflection points, and ${\theta}_{\mathrm{r}}$ is the changing deflection angle between the two reflected beams. It is worth noting that the equation only involves the relative changes between two beams, and this design has a relatively weak anti-interference ability. To further improve the anti-interference ability and measurement accuracy, in practical applications, usually more than two parallel beams are adopted, and the random error is reduced by averaging the measurement values of adjacent beam pairs. With the high sensitivity and stability brought by this multi-beam geometric design, the MOSS method has become one of the preferred techniques for in situ monitoring of stress and strain for LIB in the laboratory [15, 56, 60, 64, 65, 77].

**Fig. 7:**
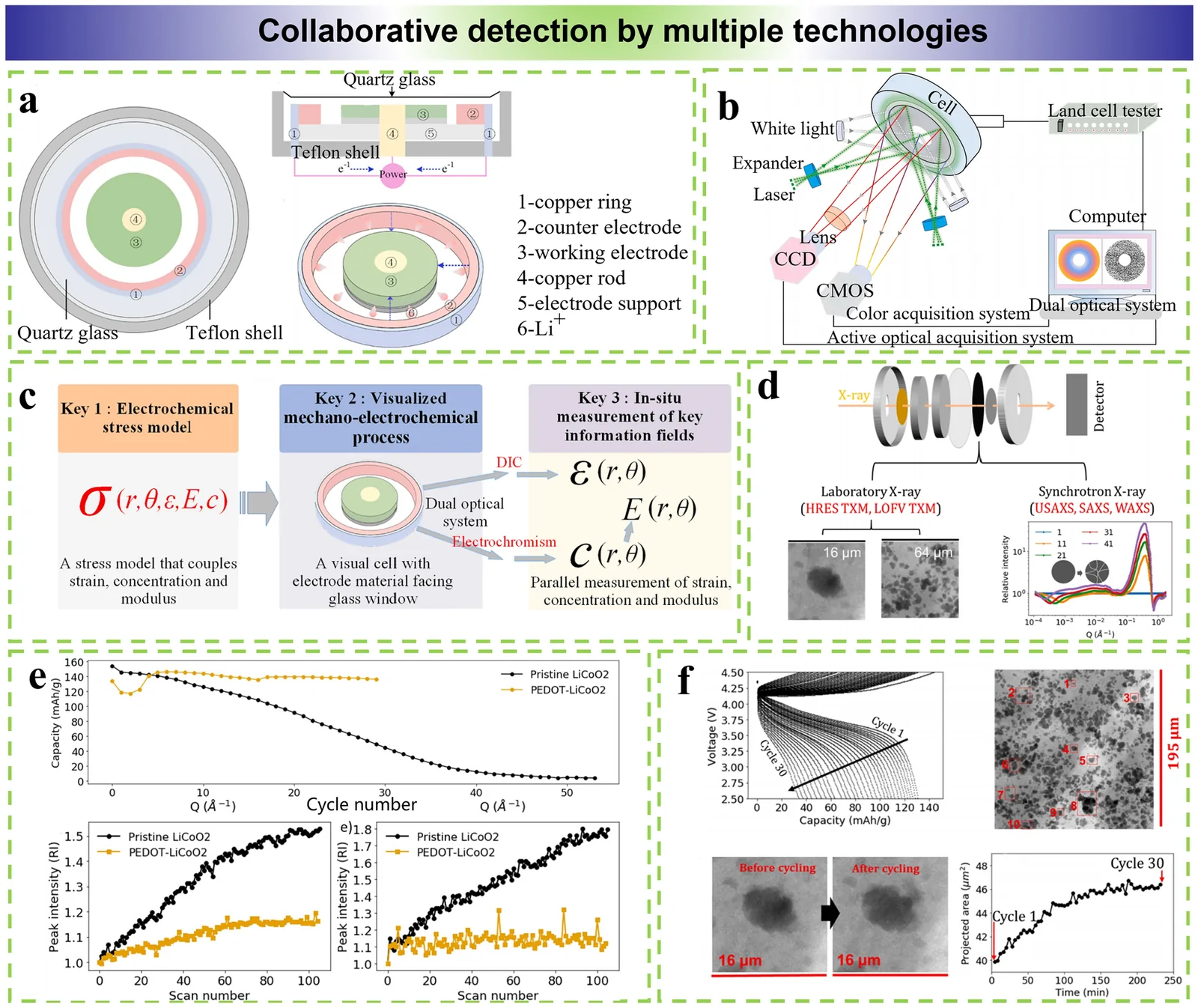
**a** Schematic diagram of the electrochemical cell structure in the dual optical system. **b** Dual optical acquisition system capable of simultaneously measuring electrode strain and Li^+^ concentration during electrochemical cycling. **c** In situ visualization method for measuring internal stress in LIBs electrodes [80]. ©2021 Pergamon-Elsevier Science Ltd. **d** Schematic diagram of the in situ analysis technique combining synchrotron X-ray scattering and high-resolution transmission X-ray microscopy. **e** Capacity changes and X-ray scattering data of LiCoO_2_ electrodes with different structures during high-voltage cycling; **f** Size changes and scanning electron images of LiCoO_2_ particles during the particle expansion process under high-voltage cycling conditions [82]. ©2021 Elsevier Science Ltd

Among them, the Mukhopadhyay team has adopted the MOSS method in multiple research works [15, 56, 60, 64, 65]. For instance, Mukhopadhyay et al. synthesized vertically aligned graphene electrodes through supramolecular methods and utilized the MOSS technique to reveal that employing crystal orientation-controlled graphene anode materials in LIBs could significantly modify the stress evolution pattern during the lithium-ion intercalation process. Most of the stress inversion occurs within the voltage range below 0.25 V, and the stress at high voltages is largely irreversible (Fig. 6d, e). Meanwhile, by monitoring the stress evolution of the graphene electrode from different directions, the high anisotropy of the graphene electrode has been confirmed (Fig. 6f, g). Moreover, the vertically oriented electrodes could achieve faster lithium-ion intercalation and deintercalation, demonstrating a higher capacity retention during high-rate cycling [15]. Pharr et al. developed a quartz window electrochemical cell that enabled the simultaneous operation of multiple working electrodes and lithium foil counter electrodes. They employed the substrate curvature technique to measure film stress, thereby establishing a functional relationship between the fracture energy of silicon thin-film electrodes and lithium concentration. This work provides valuable insights into the fracture mechanisms and structural design considerations for silicon electrodes [79]. In practical applications, the MOSS method is usually used in combination with other technologies. For example, Xie et al. developed a dual-path acquisition system (Fig. 7a) that enables the simultaneous measurement of in situ strain fields and Li^+^ concentration distributions. This multi-physics approach facilitated, for the first time, a quantitative characterization of the dynamic evolution of the electrochemical stress field (Fig. 7b, c) [80]. Although the changes in electrode stress can be obtained in real time, it is still impossible to establish the intrinsic connection between the morphological changes of electrode materials and stress. In addition, there are many similar research methods. Su et al. combined synchrotron X-ray scattering technology with transmission X-ray microscopy (TXM) technology (Fig. 7d) to obtain cross-scale spatial information and explored the irreversible phase transformation and high-pressure cycling failure mechanism during the lithiation process of LiCoO_2_ (Fig. 7e, f) [81]. It can effectively characterize the morphological evolution process of the electrode, but can only obtain information related to stress and strain indirectly.

Generally, the LBPD method is straightforward to implement; however, it is primarily suitable for analyzing simple stress fields. This approach can only capture local stress and is inadequate for characterizing complex stress fields. Although the MOSS measurement method exhibits a certain degree of resistance to vibration interference, relying solely on a single in situ measurement technique is inadequate for a comprehensive analysis of the stress evolution and degradation mechanisms of the mechanical properties in the internal electrode materials of LIBs (summarized in Table 1**)**.

**Table 1:** Advantages and limitations of the two optical detection methods

Method	Advantage	Limitations	Application scenario
MOSS	Real-time quantitative stress monitoring (Stoney formula) Resistant to vibration interference, relying on the change in the relative spacing of the light beams	The base active substance stiffness matching must be precise, and the back needs to be polished The porous electrode requires a revised stress model	Mechanical failure mechanism of high-capacity electrodes Interface/buffer layer optimizations Phase transition and the mechanical contribution of SEI Stress–electrochemical coupling modeling
LBPD	The real-time monitoring of stress changes can also monitor electrode size changes	Single-beam measurement is susceptible to interference: Environmental vibration can easily lead to the loss of measurement reference points, thereby causing significant errors	It is mainly used in electrochemical stiffness studies that require simultaneous measurement of stress and strain, actual stress analysis of porous composite electrodes, as well as interface mechanics of solid-state batteries and metal negative electrodes

## In Situ Acoustic Detection

In situ acoustic detection uniquely overcomes the fundamental challenges associated with many other real-time monitoring techniques. By providing a noninvasive and highly penetrating probe, it circumvents the need for compromising the cell hermetic or implanting performance-altering sensors, making it a powerful candidate for investigating internal stress and strain of LIBs. Its unique penetrating capabilities make it particularly suitable for this application. By monitoring the changes in the propagation characteristics of sound waves during the charging and discharging processes of the battery, it is possible to deeply analyze the capacity attenuation mechanism, structural evolution, and health status inside the LIBs. In the field of in situ acoustic detection of LIBs, it mainly involves two major categories: ultrasonic (US) detection technology and acoustic emission (AE) detection technology.

### Ultrasonic Wave Detection Technology

US detection technology mainly works by emitting high-frequency US waves to LIBs and assessing and monitoring their status based on the reflection, transmission, and attenuation characteristics of US waves as they propagate through the multilayer battery structure. The testing device requires two acoustic probes: one for emitting US waves and the other for receiving them. Its working principle is based on the change in acoustic parameters caused by volume change of materials: When lithium ions are inserted or extracted during charging and discharging, the structural changes of electrode materials will alter the length of the US wave propagation path. By precisely measuring the change in the time of flight (TOF) of US waves, the state of charge (SOC) can be calculated [83]. Meanwhile, structural damage caused by electrode aging will lead to a reduction in US wave amplitude and changes in frequency. By analyzing the time domain and frequency-domain characteristics of the signal, the state of health (SOH) can be evaluated (Fig. 9a-c).

Due to the mechanical deformation of the internal materials in LIBs during the cycling, the propagation speed and signal amplitude of the US signal will also change. The signal propagation speed is measured by the time difference between the emission time and the reception time, which is called the TOF, and the correlation between the input and output signals is analyzed through Eq. (6) [84]:

6 $$[f * g](\tau ) = - \int_{ + \infty }^{\infty } {f(t)} g(t + \tau )dt$$

Among them, f is the transmitted signal, g is the received signal, τ is the relaxation signal, and *t* is the time. Another method is to detect the structural changes of the material by comparing the variations in signal amplitude A, as shown in Eq. (7):

7 $$A = \int_{ti}^{tf} {\left. {\left| {f(t)} \right.} \right|} dt$$

Among them, tf and ti are used to limit the measurement waveform range. The transmission signal strength is measured, and the variation results under different states are compared. At the same time, combined with other characterization methods, the changes in the material structure are judged.

**Fig. 8:**
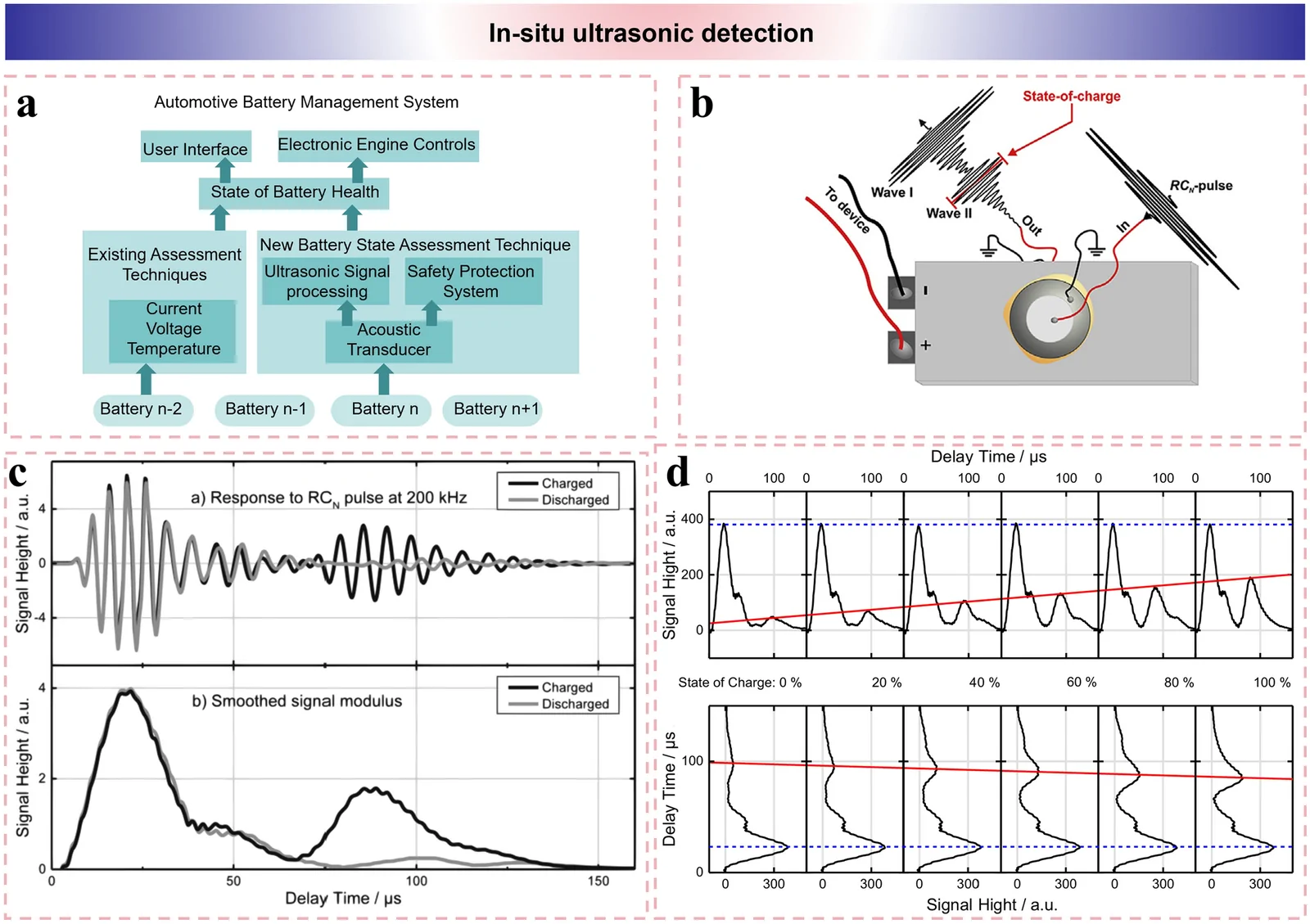
**a** Schematic diagram of the battery health status assessment and management system based on ultrasonic transducers [86]. ©2018 Elsevier. **b** Schematic diagram of the ultrasonic detection device for detecting SOC by applying cosine pulses; Pulse responses of the pouch LIB **c** at fully charged and fully discharged states and **d** delay changes of the LIB at different SOC levels [87]. ©2017 Elsevier

The application of US techniques for in situ LIB monitoring was pioneered by Redko et al. in a seminal 2010 patent that outlined an apparatus and method for evaluating the service life of LIBs [85]. The transmitting probe array and the receiving probe array are located on both sides of the battery. The probes are positioned through the mutual inductance phenomenon of the coils. According to the voltage signal of the induction coil, the position of the transmitting array is adjusted to ensure that all probes are aligned, thereby optimizing signal transmission. The signal distribution is analyzed through a three-dimensional regression surface, and the average value or extreme value is calculated. This reliance on the calibration curve to determine remaining life ignores a crucial reality of battery degradation: the aging process is not a uniform, linear progression. Instead, it is a highly path-dependent phenomenon, profoundly influenced by the unique operating environment and usage conditions. Notably, there are discrepancies between the degradation patterns observed during the calibration process and those encountered in actual applications. This complex individual difference poses challenges to the universality and accuracy of the existing aging prediction models. In response to the above issues, Sood et al. proposed an US detection technique based on acoustic impedance changes to determine the state of health (SOH) of batteries (Fig. 8a). By analyzing the amplitude of reflected and transmitted ultrasonic waves, this method provides a dual-capability diagnostic. It can detect bulk changes indicative of SOH, such as structural degradation or gas evolution, while also using the reflected signals to spatially resolve and map specific areas of internal damages. However, this method is mainly applicable to large-volume commercial pouch LIBs and is relatively costly [86]. Gold et al. addressed the issue of high detection costs of the US method in the in situ stress–strain detection for LIBs and introduced a new type of US transmission probe. They used piezoelectric buzzers to conduct in situ tests on the graphite anodes of LIBs. By adhering the sensors to both sides of the battery with epoxy resin adhesive (Fig. 8b) and applying cosine pulses to the interior, this pulse signal is highly sensitive to the pore changes in the graphite anode during the charging/discharging process. By establishing a linear correlation between the observed US pulse signals and the SOC, it can be used to predict the SOC of LIBs during the charging and discharging process (Fig. 8c, d). This method is particularly suitable for large-scale quality control and troubleshooting in commercial LIBs manufacturing, characterized by its advantages of low power consumption, independence from electrical measurements, and a compact, lightweight sensor design [87].

**Fig. 9:**
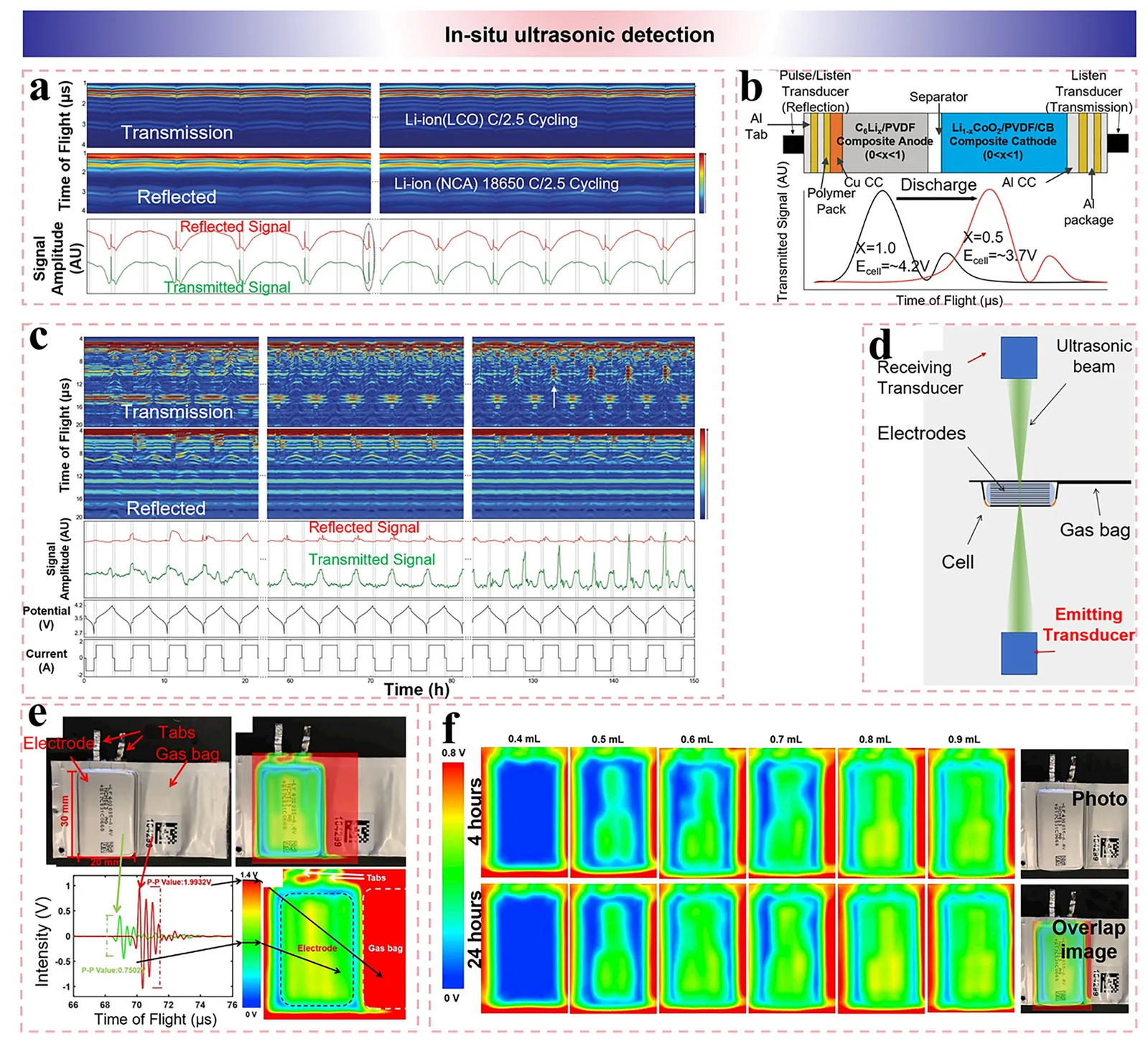
**a** TOF spectra of LiCoO_2_/graphite cylindrical cells in transmission and reflection modes. **b** Schematic diagram of the cylindrical cell detection device, showing that TOF increases with the change of SOC during the discharge process. **c** TOF spectra and signal amplitude changes of the cylindrical cell within the first 1–34 cycles [83]. ©2015 Royal Soc Chemistry. **d** Schematic diagram of the path of the focused ultrasonic beam. **e** Ultrasonic transmission images of NMC532/graphite pouch cells and the changes in transmission waves at different positions. **f** Ultrasonic transmission images of pouch LIB at different times after being wetted by the electrolyte [31]. ©2020 Cell Press

Based on the correlation between signal amplitude and the TOF of the battery to SOC and cycle life, researchers have extended the US method to predict the end of battery life based on previous studies [88]. Most previous studies have focused on pouch batteries, while Oca et al. further applied this method to cylindrical batteries [89]. Moreover, this technology is no longer limited to evaluating the operating state of the battery by comparing the changes in US signals between new and aged batteries. For example, Zhang et al. used an ultrasonic defect detector (Olympus Corp, Japan) to study the kinetic evolution of NCM622 cathodes and graphite anodes at different drying temperatures, thereby discarding the simulation of drying in previous studies and providing valuable guidance for optimizing the drying process in electrode manufacturing [90]. Deng et al. developed a nondestructive and highly sensitive ultrasonic imaging technique, which can visually reflect the distribution state of the electrolyte inside the battery by converting the local transmission rate of ultrasonic waves in pouch batteries into color images (Fig. 9d–f), providing a new method for revealing the failure mechanism of lithium-ion pouch batteries [31]. Besides, compared with X-ray and neutron imaging techniques, this method offers greater operational simplicity and superior sensitivity to changes in the electrolyte and internal gas evolution.

Beyond correlating acoustic parameters with SOC and SOH, it is important to recognize the underlying physical connection between ultrasonic wave propagation and the mechanical stress state of electrode materials. The velocity of an ultrasonic wave in a solid medium is governed by the material’s elastic modulus and density. During lithiation/delithiation, the substantial changes in the electrode’s elastic modulus—as demonstrated by Li et al. who observed a decrease from ~ 0.63 to ~ 0.18 GPa in a composite Si electrode—directly affect the acoustic wave velocity and, consequently [68], the TOF. From the perspective of acoustoelastic theory, the stress state within a material modifies its effective elastic constants, thereby altering wave propagation characteristics. Thus, TOF variations inherently encode information about the evolving stress field within the electrode, although extracting quantitative stress values from TOF signals alone remains challenging and typically requires complementary calibration against independent stress or strain measurements.

### Acoustic Emission Detection Technology

**Fig. 10:**
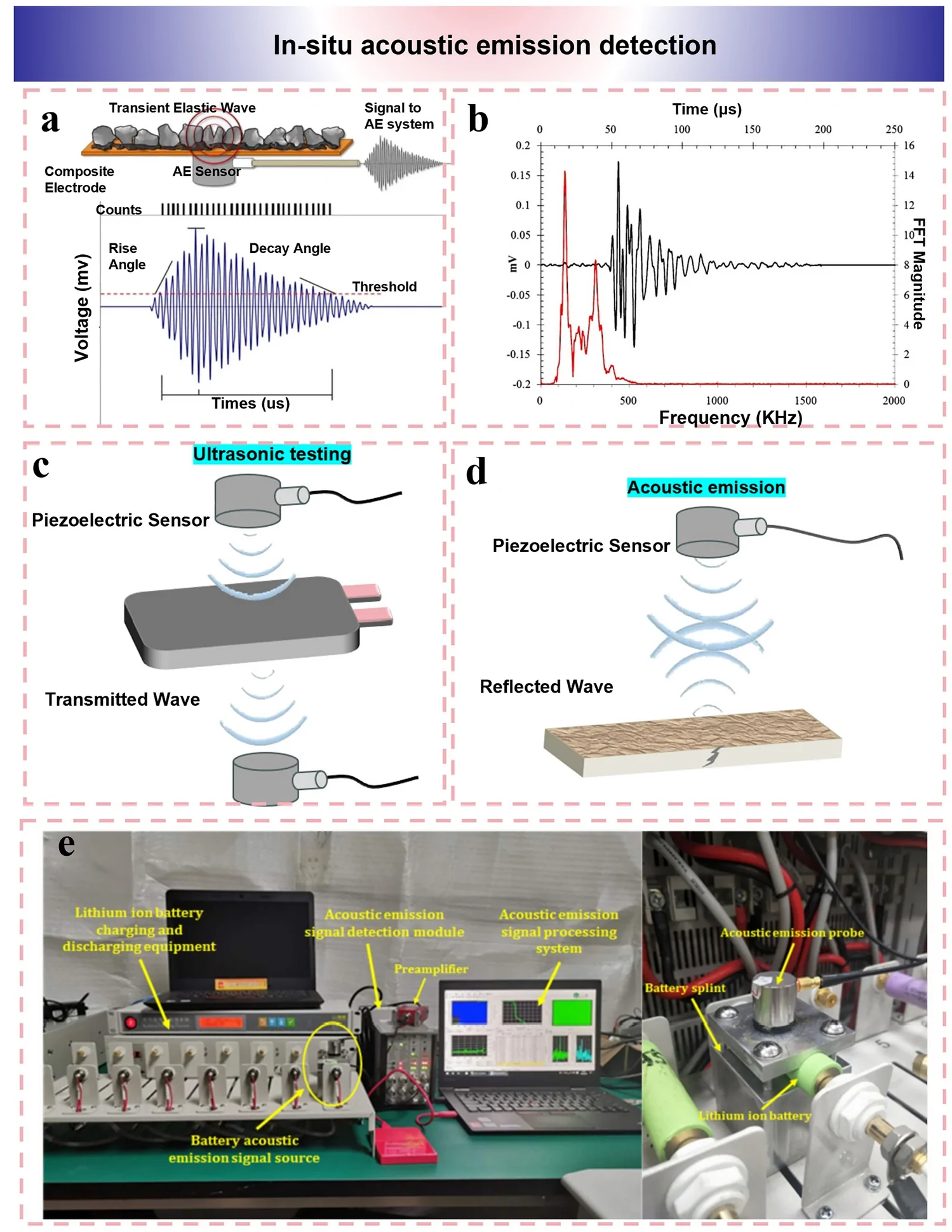
**a** Process by which electrode fracture generates acoustic signals and reaches the acoustic sensor, as well as typical acoustic signals. **b** Typical acoustic wave signals during the cycling process of LIB silicon electrodes [97]. ©2010 Electrochemical Soc Inc. **c** Ultrasonic testing, **d** working principle of acoustic emission technology, and **e** schematic diagram of acoustic emission experimental device [91]. ©2021 MDPI

Acoustic Emission testing technology is a nondestructive testing method based on mechanical properties. During the charging and discharging process of LIB, the deformation, phase transition, or micro-crack expansion of the internal materials will lead to rapid energy release and generate stress waves (Fig. 10a, b). By collecting and analyzing these stress wave signals with acoustic probes, the internal state of the LIBs can be monitored. These AE serve as direct signatures of irreversible chemo-mechanical events within batteries, such as the fracture of electrode particles or the dynamic growth and rupture of the SEI.

Compared with US method, although both are based on the interaction between sound waves and materials to obtain internal information of the battery, US detection is an active detection (Fig. 10d), while AE detection is a passive detection (Fig. 10c). AE signals from LIBs are classified as either continuous or pulse signals based on their generation mechanism. Continuous AE signals, characterized by a persistent, wavelike form, are primarily linked to ongoing electrochemical side reactions such as gas generation during cycling. Pulse AE signals mainly originate from the rapid expansion of internal cracks in electrode materials and are directly related to the cracking and aging of electrodes during the cycling process [91, 92]. Recently, the application of AE technology has proven to be a powerful tool for the in situ investigation of mechanical failure in high-capacity electrode materials (Fig. 10e), such as graphite anodes [93], silicon anodes [94–97], and lithium iron phosphate cathodes [98]. These studies have strongly confirmed the effectiveness of AE technology in achieving in situ detection of LIBs and exploring its failure mechanism. In early studies, Ohzuku et al. used this method to detect the cracking behavior of lithium manganese oxide electrodes during electrochemical cycling [98, 99]. Thanks to the excellent penetration ability of acoustic signals, AE technology is not only applicable to button cell research but also to pouch cell systems. Komagata et al. applied AE technology to detect commercial 18650 full cells and found that the AE signals generated during cycling mainly originated from the crack propagation caused by phase transitions of electrode materials. Notably, their research observed that AE signals could be detected in the early stage of charging, while significant AE signals could only be detected under high-rate conditions during discharging [100]. Currently, AE technology is also gradually being applied to SOC prediction [97] and the study of unstable phenomena caused by SEI [99, 101]. For instance, Fordham et al. collected and analyzed AE signals from 18650 cylindrical LIBs during the cycling aging process, obtaining the AE signal threshold for battery aging. They also combined machine learning to establish the correlation between AE signals and performance degradation, developing an efficient and cost-effective method for predicting the SOH of cylindrical LIBs during cycling [102]. Wang et al. also utilized AE technology to summarize the changes in AE signal amplitude and energy during the capacity decay process of 18650 cylindrical LIB [103].

From a stress detection perspective, AE offers a fundamentally distinct yet complementary modality compared to optical and electrical methods. While MOSS and strain gauges provide continuous, real-time curves of average stress or strain, AE captures the discrete, irreversible stress relief events that occur when local stress concentrations exceed the fracture strength of electrode materials. Each AE “hit” is therefore a direct signature of a stress-driven failure event at the microscopic scale. Quantifiable AE parameters—such as cumulative event count, hit amplitude, rise time, and frequency centroid—serve as statistical indicators of damage accumulation rate and failure mode. For instance, the number of AE events per cycle has been shown to correlate strongly with capacity fade in Si-based electrodes [97], while the frequency content of AE signals can distinguish between particle cracking (high-frequency) and gas bubble formation or SEI rupture (lower-frequency) [91]. This event-based, damage-centric perspective makes AE uniquely suited for detecting and characterizing the specific stress behaviors that optical curvature methods cannot access, such as localized particle fracture and interfacial debonding within thick, porous composite electrodes.

Acoustic detection technology is highly favored due to its low cost, non-intrusiveness, and high sensitivity. More importantly, most electrode materials will generate detectable acoustic signals during the process of performance degradation. This enables acoustic detection technology to provide in situ dynamic information about electrode material damage, which not only helps to accurately locate damage but also provides key data for evaluating the modification effect of the mechanical properties of electrode materials. However, it still faces the problem of difficulty in transitioning from laboratory research. The key bottleneck for commercialization lies in how to expand from single-cell detection to the detection of battery module or battery pack levels. This leap involves many complex issues: the acoustic responses of different materials under different working conditions; the complex data processing and analysis of concurrent signals from multiple battery cells; and the separation of effective signals from interference signals generated by adjacent battery cells. The advantages and limitations of the two acoustic detection methods are shown in Table 2.

**Table 2:** Advantages and limitations of the two acoustic detection methods

Method	Advantage	Limitations	Application scenario
AE (Passive detection)	Real-time capture of microscopic failure events: particle cracking, SEI rupture, lithium exsolution, gas production No external incentives required High temporal resolution, capable of tracking transient processes	Multiple-source signal aliasing occurs, making it difficult to identify the source of failure The environment is highly sensitive to noise and strict vibration isolation requirements are imposed	Monitoring of crack propagation and particle pulverization in electrode materials Safety warnings for excessive charging/overdischarging and other abusive usage conditions Gas production and interface stratification detection Real-time estimation of SOC/SOH
US (Active detection)	Directly characterize overall physical properties such as density, modulus, and interface contact Highly sensitive to gas production and electrolyte infiltration	Dependence on coupling agents limits long-term stability The selection of frequency determines the sensitivity of the defect detection; the parameters depend on the specific system	Visualization of the electrolyte infiltration process and defect screening Gas production and interface stratification detection Real-time estimation of SOC/SOH

It is instructive to compare the stress/strain quantification pathways of acoustic methods with those of optical and electrical approaches. Optical methods (MOSS, LBPD) rely on the film–substrate curvature principle and Stoney’s equation to convert curvature changes into average film stress. Electrical methods (strain gauges) exploit the piezoresistive effect and Hooke’s law to map strain to stress. In contrast, acoustic methods establish stress state correlations through two distinct routes: (1) active ultrasonic detection, which probes the acoustoelastic effect whereby stress-induced changes in elastic modulus alter wave propagation velocity and attenuation; and (2) passive acoustic emission, which registers the elastic waves generated by stress relief events themselves. While the acoustic routes do not yield direct, real-time stress–strain curves with the same immediacy as optical or electrical techniques, they offer unique advantages: deep volumetric penetration through multilayer cell structures, high sensitivity to the earliest stages of mechanical degradation, and the ability to detect stress-driven phenomena (such as internal particle cracking and gas evolution) that are invisible to surface curvature or strain-based methods. The integration of acoustic detection with complementary optical or electrical techniques therefore represents a promising direction for achieving comprehensive, multi-dimensional stress characterization in both laboratory and commercial battery systems.

## In Situ Electrical Detection

Real-time in situ monitoring of internal electrode states, a critical requirement for ensuring the safe commercial application of LIBs through adaptive charging strategies and risk assessment, has long been a significant challenge. In this context, in situ electrical detection methods offer a compelling combination of advantages. These methods are founded on the linear correlation between the resistance of a strain-sensitive material and its mechanical deformation. Their key benefits include: (1) Ensure high measurement accuracy while maintaining the operational conditions of the battery, thereby facilitating real-time monitoring of the dynamic evolution processes occurring within internal electrode materials; (2) exhibit low sensitivity to environmental interference and demonstrate robust performance under varying environmental conditions; (3) allow for straightforward data processing, with a single system capable of supporting multi-channel parallel detection, thus enabling synchronous monitoring of battery modules; and (4) feature low cost and ease of operation, presenting significant potential for commercial application.

### Resistance Strain Sensor

**Fig. 11:**
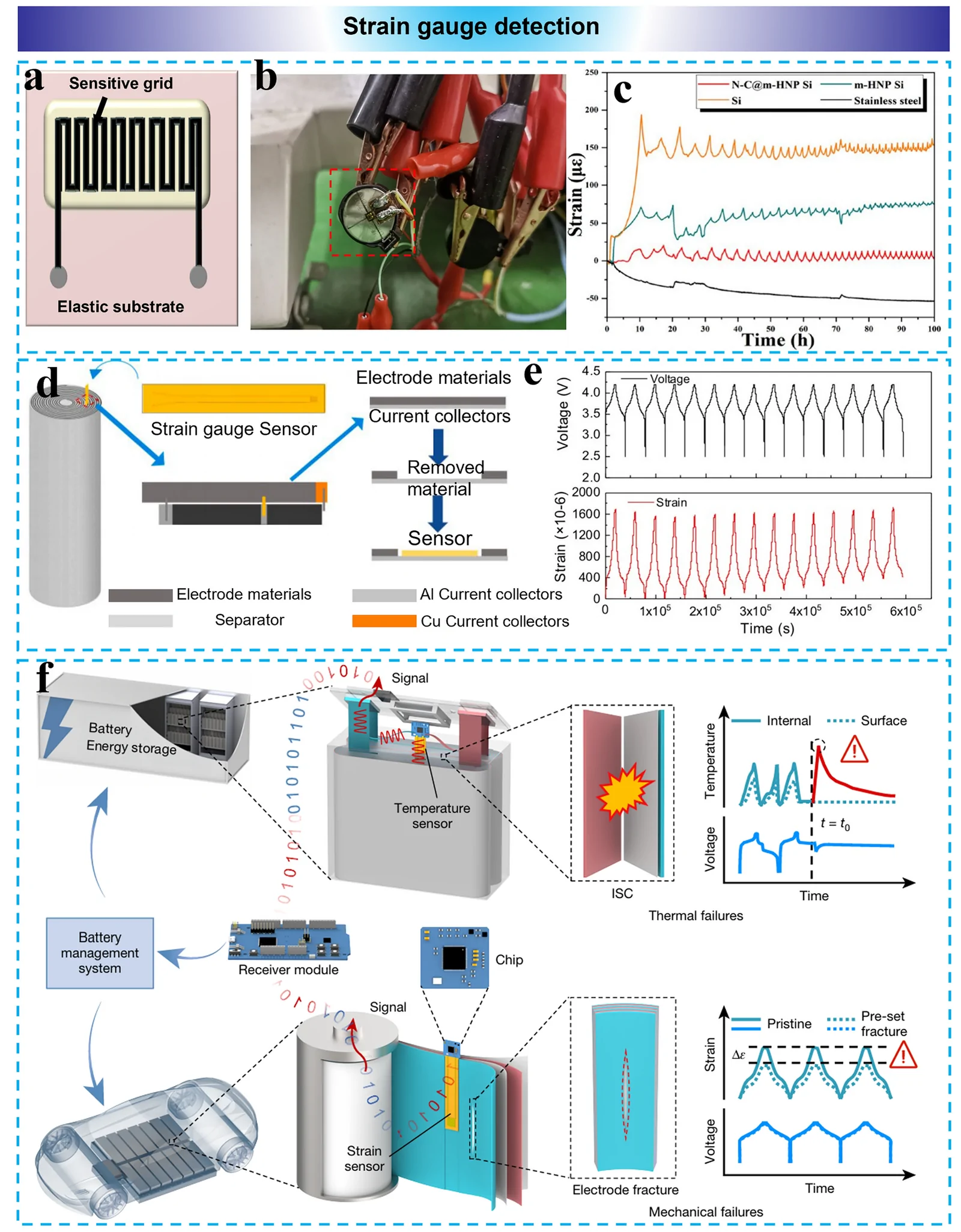
**a** Schematic diagram of the resistance strain gauge structure; **b** Schematic diagram of the button cell strain measurement device using the resistance strain gauge. **c** Strain variation over time of different silicon anodes during the long cycle process [105]. ©2024 Amer Chemical Soc. **d** Schematic diagram of the in situ measurement device for 18650 LIBs and the process of integrating thin-film strain sensors; **e** Strain variation over time of 18650 LIBs within 15 cycle periods [106]. ©2021 Elsevier. **f** Working schematic diagram of the strain and temperature sensors implanted inside the LIBs and the detection mode of thermal runaway and mechanical failure inside the battery [34]. ©2025 Nature Portfolio

Resistance strain sensor provide a direct electrical route to in situ strain measurement in LIBs. The working principle of resistance strain gauges is mainly piezoresistive effect, that is, the mechanical strain is directly converted into a change in resistance. A typical gauge (Fig. 11a) comprises a sensitive metallic grid on a compliant polymer substrate. When bonded to a surface of batteries, this assembly ensures that any strain from internal stress-induced deformation is faithfully transferred to the grid, thus modulating its electrical resistance. This transmission results in alterations to its geometric dimensions and resistivity, consequently affecting its overall resistance. Based on Ohm’s law, a linear relationship between resistivity ΔR/R and strain ${\boldsymbol{\varepsilon}}$ is derived [104]:

8 $$\frac{\Delta R}{R} = \kappa \cdot \varepsilon$$

9 $$\kappa = \frac{d\rho }{\rho } + (1 + 2\mu )$$

Among them, R represents the resistance of the metal resistance wire, κ is the strain sensitivity coefficient of the resistance wire, ρ is the resistivity, and μ is Poisson’s ratio. In actual measurement, a Wheatstone bridge circuit is needed to convert the micro-strain $\mu \varepsilon$ into an easily processed voltage signal. Combined with the material constitutive relationship (Hooke’s law: $\sigma =E\varepsilon$, where σ is the stress value and E is the Young’s modulus), a quantitative mapping relationship between the strain signal and the internal stress can be established to achieve real-time monitoring of stress evolution.

Resistive strain gauges offer several notable advantages, including compact size, lightweight design, high sensitivity, and rapid response times. These characteristics make them particularly suitable for in situ studies of the mechanical behavior of small- to medium-sized LIBs, such as button cells and 18650-type LIBs. In recent years, researchers have successfully applied them to various battery systems. Dai et al. established a mechanical model of the single-sided positive electrode shell of a button cell based on the thin plate small deflection theory in elasticity mechanics. By using foil strain gauges to monitor the strain at the center of the positive electrode shell in real time (Fig. 11b), they derived the real-time evolution of internal stress through formula (10) and evaluated the modification effect of porous silicon anodes using this method (Fig. 11c) [105].

10 $$q_{0} = \frac{{8Et^{2} }}{{3(1 - \mu )(3 - 3\frac{{b^{2} }}{{a^{2} }} + \frac{{b^{4} }}{{a^{4} }})\frac{{b^{2} }}{{a^{2} }}\left[ {a^{2} (1 + \mu )} \right]}}\varepsilon$$

**Fig. 12:**
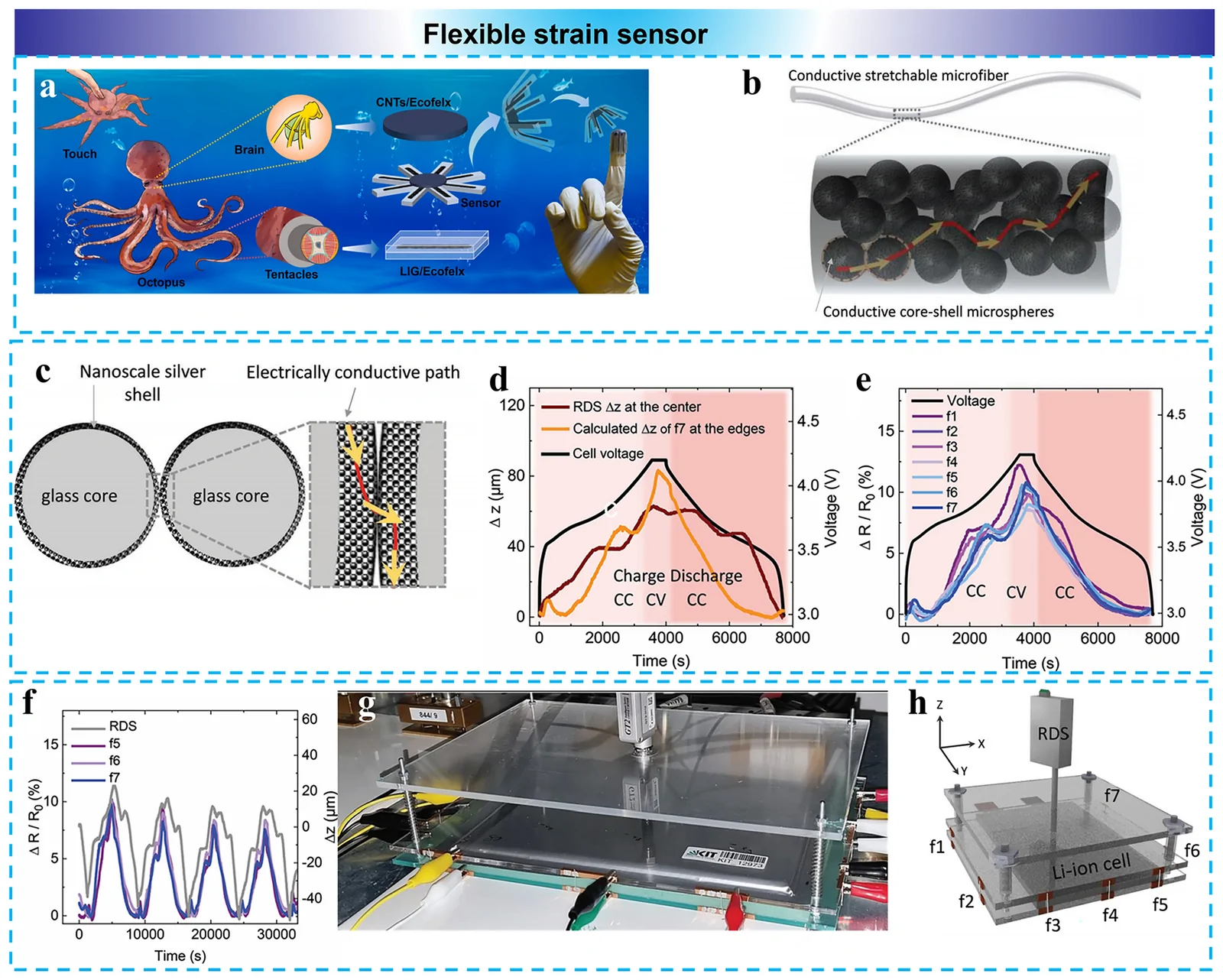
**a** Bionic sensor imitating the tactile ability of octopus [107]. ©2023 Wiley-V C H Verlag Gmbh. **b** Schematic diagram of the fiber material structure. **c** As pressure rises, the interconnectivity of spherical particles decreases, leading to reduced electrical conductivity. **d** During one charge and discharge cycle, the red curve represents the thickness change of the lithium-ion battery center position in the Z direction as monitored by the reference sensor. The yellow curve shows the displacement change curve caused by the tensile strain of the microfiber sensors f1–f7 due to the thickness change of the lithium-ion battery. The black curve represents the voltage change curve of the battery. **e** Resistance variation curve caused by tensile strain and the voltage–time variation curve of the battery are generated by the microfiber sensors f1–f7. **f** Relative resistance changes of three microfiber sensors during four consecutive charge–discharge cycles and the thickness variation curves of the battery center position in the Z direction monitored by the reference sensor. **g** Photo of the bag battery thickness monitoring device. **h** Schematic diagram of bag battery thickness monitoring device. To measure the thickness change in the Z direction of the bag battery, the response data of the fiber strain sensors f1 to f7 are simultaneously collected through multiple channels [33]. ©2024 Elsevier

Among these parameters, ${q}_{0}$ represents the internal pressure exerted on the positive electrode shell of the button battery; E denotes Young’s modulus; *t* indicates the thickness of the positive electrode shell; μ refers to Poisson’s ratio; *a* signifies the radius of the positive electrode shell; *b* corresponds to the radius of the electrode sheet; and ε describes the strain at the center position of the positive electrode shell. Zhu et al. proposed an integration method for internal strain sensors in 18650-type LIBs (Fig. 11d, e), and for the first time, used insert-type strain gauges to measure the strain caused by internal expansion in cylindrical LIBs [106]. It is noteworthy that Fan et al. successfully integrated thin-film sensors into LIBs, enabling the conversion of varying signals into electromagnetic waves through advanced chip technology (Fig. 11f). This innovation facilitated the wireless transmission of strain and temperature change signals within the battery. This technology is of significant importance for fault location and safety warning in the design process of next-generation smart batteries [34]. Strain-sensing technology, which leverages the variable resistance effect of materials, has achieved significant advancements in material composition, structural integrity, and functional design. This progress is particularly notable in the domains of flexible wearable electronics and human–machine interaction [107–109]. These innovations have also contributed to the field of battery research; researchers have developed stretchable polymer composites and integrated them into strain sensors (Fig. 12a). Nazari et al. developed a flexible fiber material that demonstrates exceptional sensitivity under stress (Fig. 12b, c). They also designed a sensor detection method utilizing this material for the real-time monitoring of lithium-ion pouch battery thickness, the measurement results of the fiber sensor are highly consistent with those of the central reference sensor, featuring high precision and excellent repeatability (Fig. 12d–f), achieving an extremely low detection limit and high durability (Fig. 12g, h) [110]. These material and structural innovations are continuously expanding the in situ monitoring capabilities of resistance strain gauges in complex LIB systems and harsh operating conditions. To overcome the limitations of traditional strain resistance sensors, such as high-temperature sensitivity and the need for an external excitation power supply, Kim et al. utilized the piezoelectric effect of III group nitride (GaN) single-crystal films. (The mechanical bending caused by the expansion of the battery results in the deformation of the non-centrosymmetric lattice, thereby directly generating polarized charges and voltage outputs on the material surface.) They attached flexible GaN piezoelectric sensors to the surface of commercial lithium-ion battery modules for in situ monitoring at both normal and high temperatures. This work demonstrated that III-N piezoelectric film sensors can effectively achieve long-term and real-time correlation monitoring of battery mechanical deformation and health status without the need for external excitation, while being resistant to high temperatures and electrochemical corrosion [109].

### In Situ Expansion Meter

It is important to clarify the scope of “optical detection” as defined in this review. The optical techniques discussed herein MOSS and LBPD are those in which light serves as the direct carrier of stress information: internal stress alters the curvature of the electrode, which in turn changes the optical path length (deflection angle or beam spot spacing), and this change in optical properties constitutes the core measurand. This definition deliberately excludes techniques that employ optical components merely as precision displacement sensors for measuring macroscopic geometric changes. A notable example is the laser scanning dilatometer, in which a laser probe measures the absolute thickness change of the cell surface. In such configurations, the laser functions as a non-contact distance gauge, equivalent in role to a linear variable differential transformer or a capacitive displacement sensor, and does not interact with the electrode material to directly sense its internal stress state. Such expansion metrology methods are therefore discussed in Sect. 5 alongside other dilatometric techniques, under the category of electrical detection.

**Fig. 13:**
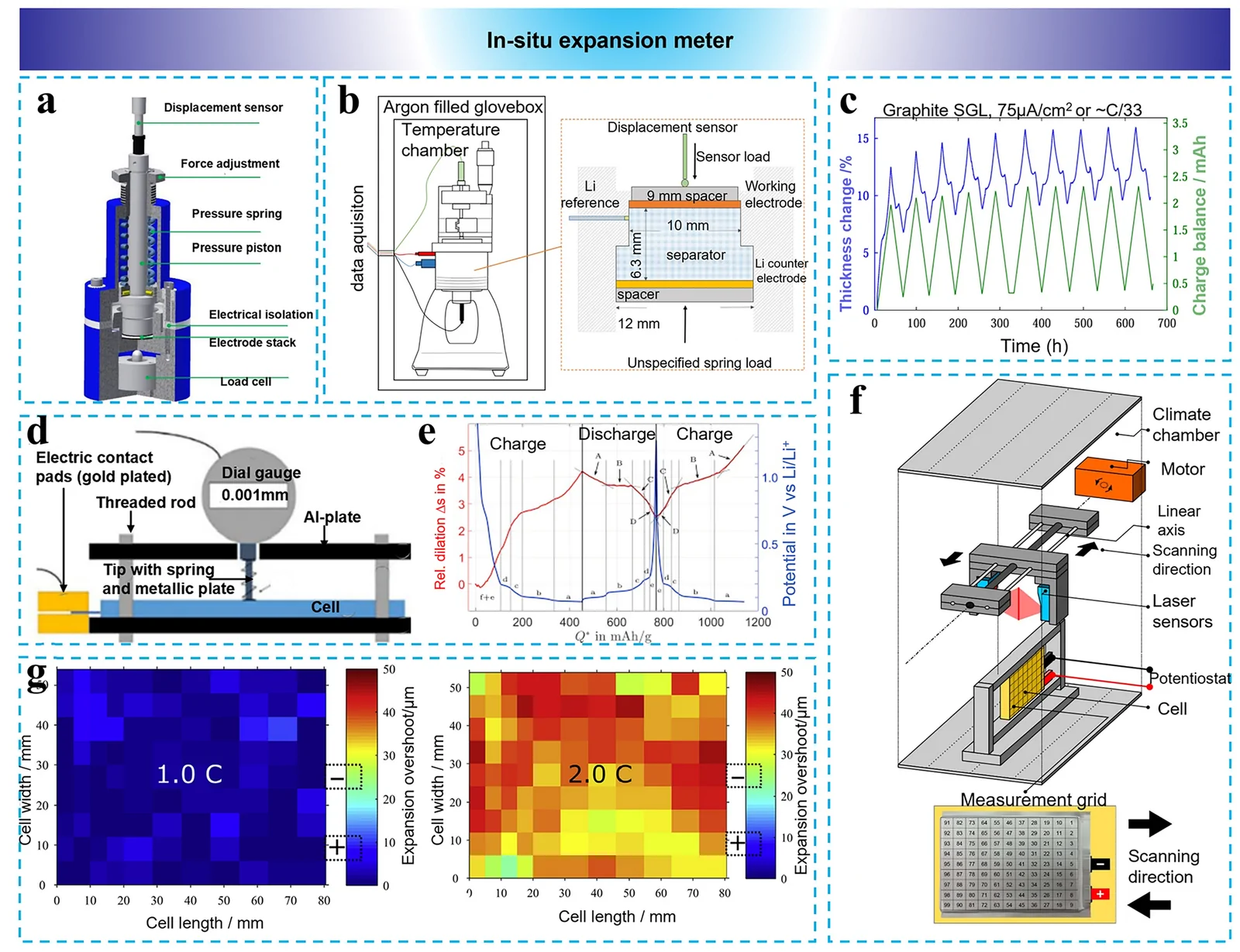
**a** Schematic diagram of the piston-type in situ expansion meter device [81]. ©2017 Elsevier. **b** ECD-3 nanoexpansion device using a capacitive displacement sensor; **c** At a current density of 75Ua cm^−2^, the changes in the thickness and charge balance of the battery over the first 10 cycles before the graphite electrode was rolled out [112]. ©2021 Electrochemical Soc Inc. **d** Expansion meter self-made by Bauer et al. The thickness change of the battery is transmitted to the LVDT through the membrane and the piston. The generated voltage output changes linearly with the battery expansion; **e** Expansion measurement of the graphite half-cell during the first half-cycle [113]. ©2018 Elsevier. **f** Schematic diagram of the two-dimensional thickness laser scanning test device; **g** Images of the expansion changes on the battery surface at different rates [114]. ©2018 Elsevier

In situ expansion meters serve as essential instruments for investigating the ion intercalation expansion behavior of layered materials, facilitating structural design, and analyzing the mechanical properties of electrode materials. According to the measurement principle, dilatometers can be mainly classified into the following types: push-rod dilatometers, piston dilatometers, and laser dilatometers. Among them, push-rod dilatometers are mainly used in the high-temperature sintering process research of solid-state battery electrolytes due to their high-temperature resistance (able to operate at temperatures above 1000 °C). In the push-rod system, the sample is placed in a high-temperature furnace, with one end fixed and the other end connected to a rigid push rod [111]. The end of the push rod is connected to a linear variable differential transformer (LVDT), which converts the mechanical displacement caused by the sample’s expansion into an electrical signal. Push-rod dilatometers are typically suitable for measuring the expansion of large samples in high-temperature environments. The two main devices inside a common piston dilatometer are LVDT and capacitive displacement sensors. The capacitive displacement sensor dilatometer infers the change in the distance between the plates based on the change in capacitance. The electrode sample is located below the piston, and the expansion and contraction of the sample push the piston to move, thereby causing a change in capacitance, from which the expansion of the sample can be calculated. This method has a high resolution. Figure 13a shows that Ivanov et al. developed an in situ expansion method for studying the macroscopic expansion of LIBs. By measuring the expansion behavior of NCM(111) cathodes and graphite anodes in full cells, they developed a theoretical method for evaluating the reversible expansion of full-cell electrodes. The research results indicated that the porosity of electrode materials mainly affects irreversible expansion and has little impact on reversible expansion [81]. Phillips et al. detected the thickness changes (The displacement of the battery surface in the vertical direction) of various high-capacity cathode and anode materials during electrochemical cycling through a capacitive displacement sensor (Fig. 13b). The research shows that for the two different types of graphite electrodes, the maximum reversible thickness changes obtained were 4.9% (for the spherical MCMB type) and 6.5% (for the sheetlike type) (Fig. 13c) [112]. This method was used to study the reversible and irreversible expansion of electrodes in the full battery structure. Bauer et al. employed a linear voltage–displacement sensor to detect the volume changes of graphite-based electrodes during cycling, developed an algorithm to reduce the expansion error caused by temperature changes, and ultimately revealed the abnormal expansion relaxation behavior of LIB graphite anodes during high-rate charge and discharge (Fig. 13d), and it is clearly pointed out that this type of expansion measurement method is highly suitable for detecting the data characteristics related to graphite classification (Fig. 13e) [113]. The laser dilatometer operates on the principles of light reflection and refraction. By shining a laser on the sample surface and detecting changes in the reflected light’s phase or position, it measures the overall expansion of LIB electrode materials across a wide range. Spingler et al. continuously measured the reversible and irreversible thickness changes on the surface of commercial pouch cells under different charging conditions using a laser scanning probe (Fig. 13f, g), and pointed out that not all excessive expansion is reversible. They ultimately proposed an efficient and safe fast charging scheme [114]. Bohn et al. developed a high-precision optical interferometric expansion measurement system, using a LED light source with a wavelength of 600 nm and a bandwidth of 20 nm. The interferometer scanned the surface of the pouch LIB with a LiCoO_2_ cathode/graphite anode at a speed of 40 µm s^−1^. Based on the difference in Z values of the collected 3D data, they achieved high-resolution, dynamic monitoring of the thickness evolution across the entire surface of a pouch cell during cycling. This approach enabled them to reveal the intrinsic correlation between battery thickness changes and its battery decay [115].

The in situ dilatometer exhibits exceptional stability in complex environments, including high temperatures and corrosive conditions, and this capability enables it to perform a comprehensive quantitative analysis of the volume expansion effects associated with electrode materials. However, it is mainly limited to providing only macroscopic overall expansion information of the electrode and are unable to capture local non-uniform deformations within the electrode in real time; resistance strain gauges are well suited for in situ mechanical monitoring of various battery types, offering simplicity in operation and ease of integration into testing systems. However, their application is limited in high-temperature environments, and prolonged exposure to corrosive conditions may compromise the long-term stability and measurement accuracy of the gauges, summarized in Table 3.

**Table 3:** Advantages and limitations of the two electrical detection methods

Method	Advantage	Limitations	Application scenario
Resistance Strain Sensor	Low cost, small size, lightweight, mature technology Flexible substrates can be easily integrated into cylindrical/supercapacitor batteries Multi-channel synchronous acquisition, supporting parallel monitoring of multiple battery packs	Sensitive to temperature, the internal non-uniform transient temperature field is difficult to be precisely compensated Adhesive thermal–hydraulic cyclic creep aging	Multi-channel parallel strain monitoring of battery pack Tracking of electrode material cyclic expansion and irreversible deformation Real-time mapping of deformation and stress for small batteries (button/18650)
In Situ Expansion Meter	Simple structure, convenient operation, and high cost-effectiveness Effectively decouple the contributions of electrochemistry and thermal expansion	Only the macroscopic average thickness is obtained, and the in-plane non-uniform deformation cannot be captured Unable to distinguish the source of irreversible strain	Measurement of the intrinsic lithiation expansion rate of electrode materials Full battery reversible/irreversible thickness change decoupling Characterization of volume expansion behavior under extreme temperatures

## Issues and Challenges

Optical, acoustic, and electrical in situ detection technologies have provided unprecedented insights into the “black box” of LIBs, revealing real-time details of internal processes ganging from electrode wetting and gas generation to phase changes and chemo-mechanical degradation. However, a critical gap persists between the rich data obtained in laboratory and the practical demands of commercial-scale application, a disparity rotted in the distinct advantages and limitations of each technique. Importantly, the transition of these in situ techniques from laboratory-scale fundamental studies to commercial battery systems introduces a distinct set of engineering requirements. Unlike idealized laboratory cells, commercial batteries—whether cylindrical (e.g., 18650), prismatic, or pouch-type—impose stringent constraints on sensor form factor, chemical compatibility, long-term durability, and non-invasiveness.

The emerging concept of the “smart batteries” promises to transcend these limitations. By integrating miniaturized, multi-functional sensors directly into commercial cells, this approach enables the acquisition of high-fidelity, multi-scale data streams in real time. When fed into cloud-based, artificial intelligence (AI)-driven digital twin models, this data can establish a powerful predictive framework for anticipating critical failure events like thermal runaway and mechanical failure. Despite this compelling vision, several urgent challenges must be addressed to bring this technology to fruition.

### Optical Detection

Curvature-based optical techniques, represented by LBPD and MOSS methods, face a set of challenges that can be systematically understood from four interconnected perspectives: the gap between simplified physical models and mechanistic cognition, environmental interference and error propagation, the chasm between ideal thin-film systems and practical porous/thick electrodes, and the ultimate challenge of synchronously decoupling stress from electrochemical signals. These challenges must be clearly recognized to properly assess the role and limitations of optical methods.

Physical model simplification and mechanistic cognition gap. The core physical basis of curvature methods is Stoney’s equation, which inherently assumes an isotropic, uniform, and defect-free elastic film perfectly bonded to a rigid substrate. In practice, composite electrodes are porous, heterogeneous, and anisotropic, consisting of randomly stacked active particles, conductive additives, and binders. Consequently, the true stress field exhibits a pronounced gradient through the electrode thickness, and the curvature measurement can only provide a spatially averaged stress value. Moreover, the measured curvature change is a convoluted response to multiple concurrent processes—lithiation/delithiation-induced strain, binder swelling, SEI growth stress, and thermal expansion—all of which are coupled into a single macroscopic bending signal. Disentangling these individual contributions remains a formidable challenge, making it difficult to directly correlate macroscopic curvature with specific microscopic failure events such as particle cracking or interfacial debonding.

Environmental interference and systematic error propagation. The operational principle of laser beam position detection is essentially a high-sensitivity optical lever, which inevitably amplifies environmental perturbations into significant stress measurement errors. In the single-beam LBPD configuration, micron-scale mechanical vibrations can lead to deviations of tens of megapascals in the calculated stress, necessitating the use of air-floating optical tables and strictly controlled inert atmospheres. Although the MOSS method achieves a certain degree of vibration resistance through its multi-beam differential design, low-frequency vibrations cannot be entirely eliminated. Furthermore, the optical windows required for in situ cells introduce additional complications—refractive index inhomogeneities, surface contamination, and gradual degradation of transmittance during prolonged electrolyte exposure all degrade the long-term stability and reliability of the optical signal.

The chasm between ideal thin-film systems and practical electrodes. Both MOSS and LBPD were developed for thin-film/substrate model systems, and their translation to commercially relevant thick-film electrodes faces fundamental obstacles. The MOSS method imposes stringent requirements: a polished back surface on the substrate, a substrate stiffness far exceeding that of the active film, and a perfect “film-on-substrate” bonding condition. Commercial battery electrodes, typically tens to hundreds of microns thick and coated on rough Cu or Al foils, are far from meeting these idealized prerequisites. Moreover, the viscoelastic time lag introduced by polymeric binders violates the purely elastic assumptions of the Stoney framework. For the LBPD method, the single-point laser measurement captures only the average curvature in the immediate vicinity of the illumination spot. In essence, optical curvature methods remain powerful “model system” tools for fundamental mechanistic investigations, but a principle-level gap separates them from the geometric, compositional, and operational realities of commercial batteries.

The key pathways for overcoming these challenges include the development of multi-beam or full-field optical techniques capable of resolving spatial heterogeneities, the introduction of advanced data fusion algorithms that integrate optical signals with simultaneous electrochemical and thermal measurements, and the synergistic combination of optical methods with high-resolution imaging and spectroscopic tools. Only through such interdisciplinary integration can the intrinsic “macroscopic measurement–microscopic mechanism” gap be effectively bridged.

### Acoustic Detection

In the acoustic monitoring of LIBs, the acoustic signals (such as US flight time, amplitude, and attenuation) are essentially a “mixed observation value” of multiple physical fields that are coupled. The core challenges of interference and decoupling in this context can be deeply understood from the following three aspects: Firstly, the nonlinear superposition and competitive contributions of multiple physical processes. Simultaneously occurring in the battery are the volume changes of electrode active materials, the continuous growth and rupture of SEI membranes, the gas evolution caused by electrolyte decomposition, and the thermal expansion and drift of sound velocity due to temperature changes. Moreover, these processes modulate the acoustic signals in different directions and amplitudes: for instance, lithiation expansion increases the material density and may reduce the sound velocity, while the appearance of gas products causes strong signal attenuation due to a sudden change in sound impedance; an increase in temperature softens the material and reduces the sound velocity, and may also accelerate the production of more gases through additional reactions. In actual operating conditions, these processes overlap and have varying time-dependent characteristics, resulting in the received acoustic response being the superposition of multiple nonlinear contributions, which cannot be distinguished through simple linear filtering or frequency-domain separation.

Second, signal interference caused by multiple reflections of the interface and wave-type conversion. LIBs have a multilayer sheet structure (current collector, electrode coating, separator, electrolyte/gas layer). Sound waves will undergo reflection, transmission and mode conversion at each interface (between longitudinal and transverse waves, and the excitation of Lamb waves). During actual battery cycling, phenomena such as the thickening of the SEI layer, local gas accumulation, and electrode stratification will dynamically change the acoustic properties of the interface, causing echoes from different depths or different regions to severely overlap in the time domain, making it difficult to track the evolution of a specific interface alone.

Third, time-varying interference from the environment and operating conditions, as well as baseline drift caused by aging. The influence of temperature on the mechanical frequency response function of the LIBs can completely mask the contribution of SOC or SOH; the Li^+^ concentration gradient generated by high-rate charging and discharging will cause local non-uniform expansion, introducing additional strain signals unrelated to the average SOC; after long-term battery cycling, irreversible volume expansion, electrolyte drying, electrode stratification, and other aging phenomena will systematically change the acoustic propagation path and attenuation characteristics, causing the acoustic reference at the same SOC to drift over time. This means that even if a high-precision data-driven model is trained under laboratory conditions, once the battery undergoes different thermal histories, different working rates or aging paths, the correlation between the acoustic features and the state relied upon by the model may fail.

The core challenge of acoustic signal interference and decoupling lies in the fact that multiple concurrent physical and chemical processes within the battery (expansion, gas production, thermal effects, aging, etc.) jointly modulate the acoustic response in a nonlinear and time-varying manner. The complex superposition of interface reflections leads to blurred signal sources, and the differences in environmental conditions and aging paths make it difficult to generalize any fixed acoustic-state mapping relationship. Therefore, how to robustly separate the target state (such as SOC or SOH) from the mixed acoustic observations and suppress the influence of other interfering factors is a key bottleneck that must be overcome for achieving acoustic-based battery state monitoring. Nevertheless, they have found a crucial niche in quality control on production lines, where they excel at rapid, batch-level defect screening to identify and eliminate substandard cells.

### Electrical Detection

Electrical detection methods are currently undergoing a transition from being regarded as “excellent laboratory diagnostic tools” to evolving into “practical embedded monitoring technologies.” Among these methods, the resistance strain gauge detection technique stands out due to its miniaturization, lightweight design, high integrability, and cost-effectiveness. By embedding these sensors within battery packs for electric vehicles and portable electronic devices, this technology facilitates real-time monitoring of the battery’s operational state, thereby enhancing the safety of future smart batteries. However, the interference and decoupling of electrical signals (voltage, current, temperature, and the resulting strain signals) present multiple complex challenges at various levels. The root cause lies in the highly nonlinear coupling of electrochemical, thermal, and mechanical processes within the battery, as well as the uneven spatial distribution.

Firstly, the interference of voltage signals mainly stems from the superposition of polarization effects, temperature gradients, and aging drift. The measured terminal voltage is the sum of the open-circuit voltage and the Ohmic polarization, concentration polarization, and electrochemical polarization. Under dynamic working conditions, these polarization components are closely related to the current direction, rate, temperature, and state of charge (SOC). The temperature gradient causes local overpotential differences, making the terminal voltage unable to reflect the precursors of local thermal runaway. For instance, Fan et al. found that the initial voltage fluctuation due to an internal preset crack was only 0.0046V, which was completely filtered out as noise in the traditional BMS (Battery Management System). Additionally, aging processes such as SEI thickening, lithium plating, and electrolyte drying alter the battery’s internal resistance and OCV–SOC curve, causing the terminal voltage at the same SOC to drift with the number of cycles.

Secondly, the interference of current signals is mainly reflected in the cumulative error of ampere-hour integration and the non-uniform current distribution dependent on the rate. The coulomb counting method requires high-precision current sensors, but in reality, there are biases, temperature drifts, and quantization errors. After long-term integration, the SOC estimation diverges severely. Although it can be corrected by OCV, the OCV requires the battery to be stationary for a long time, which is almost infeasible in actual working conditions. More importantly, at high rates, the Li^+^ concentration gradient causes different local reaction progress in the electrodes, making the measured total current unable to represent the real reaction current in each internal region. In addition, in cylindrical batteries, the electrochemical-induced strain (caused by Li^+^ intercalation) is dominant, while in prismatic batteries, thermal strain is dominant—this means that the same current amplitude contributes completely differently to the internal mechanical state in different packaging forms, and it is impossible to decouple these two contributions solely based on the current.

Thirdly, the engineering interference introduced by signal transmission and packaging cannot be ignored. Intrusive or implantable sensors (such as foil strain gauges and temperature sensors) can provide real-time monitoring of the internal state of batteries, but they also have non-negligible impacts on the internal environment, energy density, and long-term electrochemical performance of the battery. Physically, sensors occupy space that would otherwise be occupied by active materials, potentially disrupting the uniformity of the core stack and introducing local stress concentrations. Chemically, the sensor packaging materials and adhesives may corrode or swell when immersed in the electrolyte for a long time, releasing metal ions that can contaminate the electrolyte and exacerbate the instability of the SEI. In terms of thermal and electric fields, the thermal conductivity differences and micro-power consumption of the sensors can locally distort the thermal field, and if the metal parts have poor contact, they may interfere with the electric field distribution and even cause micro-short circuits. In terms of electrochemical performance, the impedance of the implanted sensors increases slightly, and the repeated expansion and contraction during cycling can lead to sensor fatigue, deadhesion, and lead breakage, and may even become a potential cause of internal short circuits. Overall, with careful design (such as ultra-thinness and optimal placement in non-active areas), these negative effects can be controlled within an acceptable range, making them more suitable for energy storage systems with lower energy density requirements but higher safety monitoring needs; for applications such as high-end electric vehicles that pursue maximum range, further miniaturization or the development of non-intrusive alternatives is still necessary.

In summary, the suitability of electrical detection methods across mainstream commercial battery formats varies considerably. For 18650 cylindrical cells, thin-film strain gauges and AE sensors have been successfully demonstrated for internal strain monitoring, though hermetic feedthrough of sensor leads remains a packaging challenge. For pouch cells, external dilatometry and ultrasonic scanning offer noninvasive alternatives well suited to the flexible, planar geometry. For prismatic hard-case cells, the rigid casing complicates both acoustic coupling and direct strain measurement, requiring sensor integration at the module level rather than the cell level. The selection of an appropriate monitoring strategy must therefore be guided by the specific battery format, the required detection fidelity, and the tolerable degree of invasiveness.

### Cross-Technique Comparative Analysis and Selection Guideline

The preceding sections have individually examined the challenges facing optical, acoustic, and electrical detection methods. However, for researchers and engineers seeking to select the most appropriate technique for a specific battery system or research question, a systematic cross-technique comparison is essential. Table 4 provides a comprehensive side-by-side comparison of the six principal methods reviewed in this work, evaluated across multiple dimensions relevant to stress/strain monitoring.

**Table 4:** Cross-technique comparison of in situ stress/strain detection methods for lithium-ion batteries

Dimension	Optical (MOSS)	Optical (LBPD)	Acoustic (US)	Acoustic (AE)	Electrical (Strain Gauge)	Electrical (expansion meter)
Physical principle	Multi-beam curvature sensing	Single-beam curvature sensing	Acoustoelastic effect (active)	Stress wave detection (passive)	Piezoresistive effect	Mechanical displacement
Directly measured quantity	Substrate curvature → film stress	Substrate curvature → film stress	TOF, signal amplitude	AE events, energy, amplitude	Strain → stress via Hooke’s law	Thickness change
Quantitative stress output	via Stoney equation	via modified Stoney equation	Indirect (requires calibration)	Semiquantitative (event statistics)	via Hooke’s law	dilation strain
Temporal resolution	Real time	Real time	Real time	Real time (event-based)	real time	Real time
Applicable electrode type	Thin film on rigid substrate	Thin film or composite on substrate	Any (noninvasive)	Any (noninvasive)	Surface-mounted (any type)	Whole cell (any type)
Commercial cell compatibility	Low (requires substrate)	Low	High (pouch, cylindrical)	High (pouch, cylindrical)	High (with surface mounting)	High (external measurement)
Invasiveness	Noninvasive (optical window)	Noninvasive (optical window)	Noninvasive (external probes)	Noninvasive (external probes)	Minimally invasive (surface)	Noninvasive (external)
Sensitivity to environment	Vibration-sensitive (partially compensated)	Highly vibration-sensitive	Moderate	Noise-sensitive	Temperature-sensitive	Temperature-sensitive
Cost	High	High	Moderate	Moderate	Low	Low–Moderate
Commercial maturity	Laboratory only	Laboratory only	Production line QC	Laboratory	Demonstrated in BMS prototypes	Laboratory, production line QC

Beyond the tabular comparison, several overarching trade-offs merit explicit discussion: Quantitative accuracy vs. practical applicability. Optical methods (particularly MOSS) provide the most direct and quantitative stress measurements, yielding real-time stress–capacity curves that can be directly compared with electrochemical data. However, this quantitative fidelity comes at the cost of stringent sample requirements—polished substrates, thin-film geometries, and optical windows—that fundamentally limit their applicability to commercial cells. Electrical methods (strain gauges and dilatometers) offer a practical middle ground, providing quantitative strain data on commercial cell formats with minimal invasiveness, though they capture only surface-averaged or net-thickness information rather than internal stress distributions. Acoustic methods, while the least direct in terms of stress quantification, offer the unique advantage of volumetric penetration through intact commercial cells, making them the only techniques capable of probing internal mechanical phenomena without any modification to the cell structure.

Active vs. passive detection philosophy. Among the six techniques, AE occupies a unique position as the only passive method: it does not excite the sample but rather “listens” for the elastic waves spontaneously generated by stress relief events within the battery. This fundamental difference means that AE is inherently an event-driven, damage-centric technique, best suited for detecting and characterizing irreversible degradation processes rather than continuously monitoring reversible stress evolution. The other five techniques are all active, providing continuous signals that track the gradual, reversible stress/strain changes accompanying normal electrochemical cycling. These two detection philosophies are highly complementary: active methods monitor the “health baseline,” while passive AE detects the “failure signatures” superimposed on that baseline.

Multimodal integration as the path forward. Given the complementary strengths and limitations summarized above, no single technique currently provides a complete picture of stress/strain evolution across all relevant scales and cell formats. The most promising direction for future research lies in the simultaneous deployment of complementary techniques. For example, combining MOSS or strain gauges (for quantitative stress curves) with AE (for damage event detection) on the same electrode could disentangle reversible chemo-mechanical stress from irreversible degradation. Similarly, pairing ultrasonic TOF measurements (for internal SOC/SOH mapping) with external dilatometry (for net expansion calibration) could provide a more complete characterization of commercial cells. Such multimodal approaches, when coupled with the AI-driven data fusion strategies discussed in Sect. 7, represent the most viable pathway toward comprehensive, real-world stress monitoring in next-generation battery systems.

For fundamental mechanistic studies on model thin-film electrodes, MOSS and LBPD remain the methods of choice due to their quantitative rigor. For commercial cell characterization and BMS integration, the choice depends on the specific objective: Electrical strain gauges are most suitable for real-time strain monitoring and early fault warning in embedded BMS applications; ultrasonic techniques excel at noninvasive SOC/SOH estimation and electrolyte wetting diagnostics; AE is preferred for detecting and classifying irreversible degradation events; and dilatometry is the method of choice for quantifying macroscopic expansion and separating reversible from irreversible contributions. For comprehensive safety–critical applications (e.g., large-format energy storage systems), a multimodal sensing strategy integrating strain, acoustic, and thermal sensors with AI-based data analytics represents the ultimate goal.

**Fig. 14:**
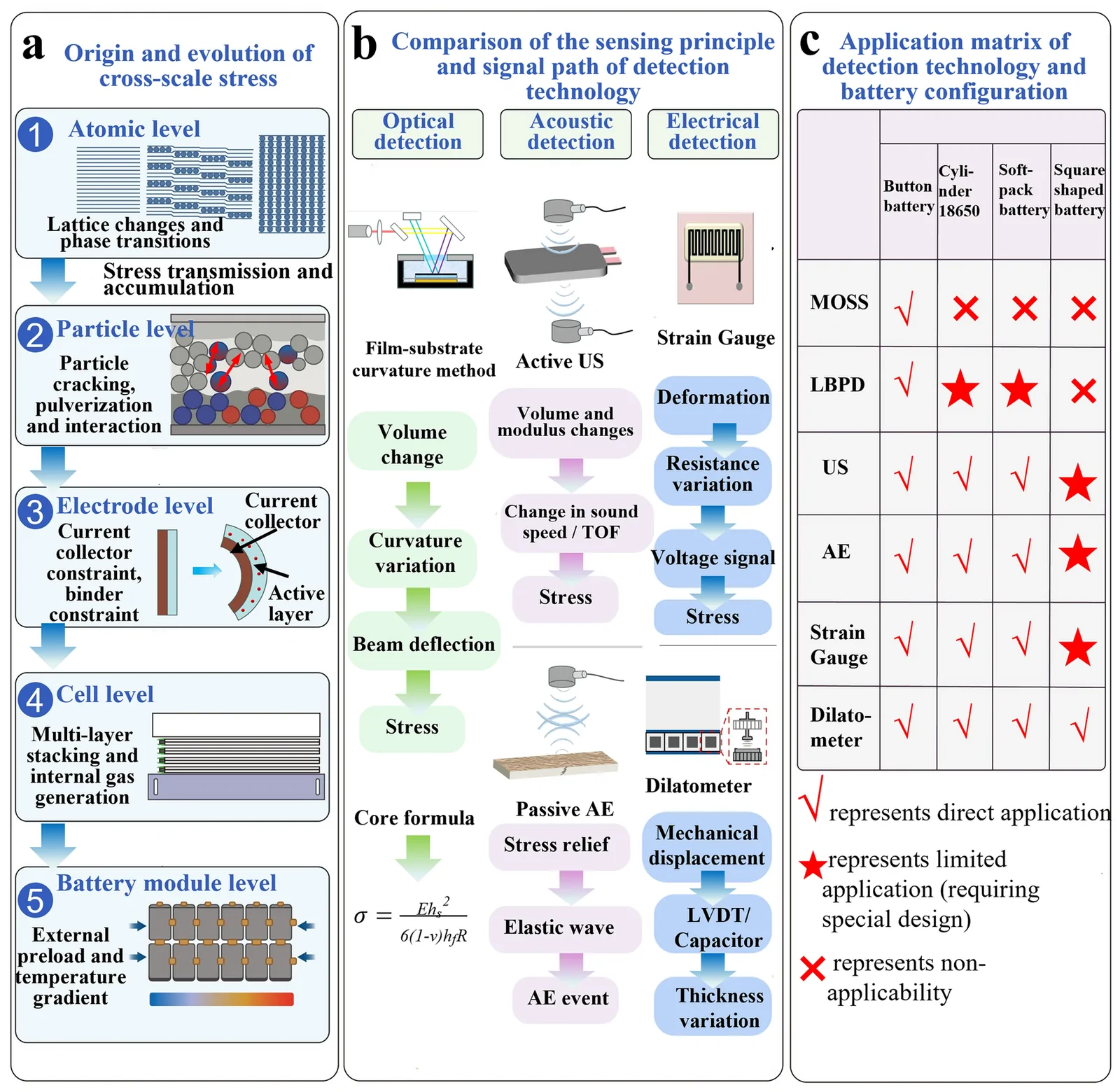
**a** Multi-scale origins and propagation pathways of stress.** b** Side-by-side comparison of the physical transduction principles and signal pathways for optical, acoustic, and electrical methods.** c** Applicability matrix mapping each technique onto mainstream commercial battery formats

To provide a holistic conceptual overview of the in situ detection landscape, Fig. 14 integrates three essential aspects of stress/strain monitoring in LIBs: (a) the multi-scale origins and propagation pathways of stress, from atomic-level lattice distortion to module-level pack swelling, (b) a side-by-side comparison of the physical transduction principles and signal pathways for optical, acoustic, and electrical methods, and (c) an applicability matrix mapping each technique onto mainstream commercial battery formats. This tripartite framework serves as both a conceptual roadmap for navigating the subsequent technical discussions and a practical selection guide for experimental design.

## Summary and Outlook

This review has systematically surveyed the state of the art in optical, acoustical, and electrical metrologies for in situ stress and strain analysis in LIBs, critically assessing their capabilities to guide future development. The insights discussed herein illuminate the pathway toward “smart batteries,” systems capable of self-sensing, self-diagnosis, and self-management that promise to enhance safety and extend the life span of next-generation energy storage.

The realization of this future is contingent upon a technological evolution from isolated, component-level measurements to integrated, system-level intelligence, driven by the fusion of flexible electronics and advanced algorithms. By embedding multi-dimensional sensors (such as potential, temperature, and pressure sensors), the battery’s internal “black box” can be opened, providing high-spatiotemporal-resolution, multi-physical data (electrochemical, thermal, and mechanical) in real time. Coupling this rich stream of sensory data with artificial intelligence will fundamentally shift battery management from passive protection to proactive, predictive control. The technical path of AI-enabled in situ detection for LIBs can be summarized as follows: Firstly, by integrating multimodal intrinsic sensing units such as flexible micro-sensors, FBG and gas sensors, and applying deep learning algorithms like convolutional neural networks for high-dimensional feature fusion, the bottleneck of single physical signals being unable to characterize complex failure processes is addressed [116–119]. Secondly, by compressing models and distilling knowledge, lightweight AI models are deployed on edge computing units to enable real-time diagnosis of thermal runaway precursors (such as early hydrogen release and sudden internal stress changes) by the onboard BMS [120, 121]. Thirdly, a physical information neural network and a “physical system–digital twin–AI engine” closed-loop framework are constructed, embedding electrochemical and thermodynamic prior knowledge into data-driven models, which not only enhances generalization capabilities but also provides interpretability to the detection results, allowing for fault pre-simulation and strategy validation in a virtual environment [122–124]. Finally, generative models such as generative adversarial networks are utilized for data augmentation of rare but catastrophic thermal runaway samples, effectively alleviating the core challenge of scarce fault data. Although concept validation studies (such as implantable three-in-one micro-sensors and reduced-order electrochemical neural network coupling methods based on single-particle models) have shown significant potential, current challenges include high costs of full-modal integration, the “black box” nature of models making it difficult to output specific failure mechanisms, long-term stability and engineering packaging of implantable devices in automotive-grade batteries [125–128]. Future research should focus on developing multi-functional integrated wireless sensing technologies, explainable artificial intelligence, and full-life-cycle digital passports to transform AI from a laboratory early warning tool into the core control engine of next-generation intelligent battery systems.
